# Dataset on flue gas composition during combustion in the fluidised bed reactor. Polyethylene combustion

**DOI:** 10.1016/j.dib.2020.106072

**Published:** 2020-07-30

**Authors:** Gabriela Berkowicz, Witold Żukowski

**Affiliations:** Faculty of Chemical Engineering and Technology, Cracow University of Technology, ul. Warszawska 24, 31-155 Cracow, Poland

**Keywords:** Flue gases composition, Polyethylene, Fluidised bed, Cenospheres, Fe_2_O_3_-cenospheres

## Abstract

The dataset presented in this article is the supplementary data for the research article titled “*The combustion of polyolefins in the inert and catalytic fluidised beds”*, in which polyethylene combustion in the cenosphere fluidised bed were investigated. The use of cenospheres as a bed material made it possible to free sink of PE particles in the bed and rule out its combustion in freeboard. It also lead to elimination of soot formation. The quantitative and qualitative analysis of flue gases were performed by Fourier Transform Infrared Spectroscopy (FTIR, Gasmet DX-4000). This data article provides detailed information on changes in product concentration at intervals of a few seconds.

**Specifications Table**

**Table:** 

**Subject**	Environmental Engineering; Chemical Engineering
**Specific subject area**	Polyethylene combustion, fluidised bed combustion, catalytic combustion
**Type of data**	Table
**How data were acquired**	Flue gases formed during thermal degradation of polyethylene in the fluidised bed were analysed by multi-component gas analyzer Gasmet DX-4000. The device incorporates a Fourier Transform Infrared Spectrometer. The Calcmet software is used as an analysis software to deconvolution of IR spectra.
**Data format**	Raw
**Parameters for data collection**	The spectra of the exhaust gas mixture were registered in the wavenumber range from 900 cm^−1^ to 4200 cm^−1^. Quantitative and qualitative decoding of each spectrum was done based on a library of reference compounds. Measurement of each spectrum and its deconvolution took 7-8 seconds.
**Description of data collection**	The bed of raw cenospheres or Fe_2_O_3_ covered cenospheres was introduced into fluidised state by a stream of air and heated to 500-900°C. Polyethylene samples were dripped from the top of the reactor to centrally fell into the bed. Polyolefin decomposition products caused changes in the composition of the gases leaving the reactor. The probe took a sample of exhaust gases from the space about 10-15 cm above the surface of the fluidised bed. Gas analyses were performed using an FTIR analyser (model DX-4000, Gasmet Technologies Oy).
**Data source location**	Cracow University of Technology, Faculty of Chemical Engineering and Technology, Cracow, Poland
**Data accessibility**	Data are accessible with the article
**Related research article**	Witold Żukowski, Gabriela Berkowicz, The combustion of polyolefins in the inert and catalytic fluidised beds, Journal of Cleaner Production, 2019, 236, 117663. 10.1016/j.jclepro.2019.117663[1]

## 1 Value of the data

The data provides detailed information on the quality and quantity of products of polyethylene degradation as a function of time.
This dataset will be helpful to develop the kinetic models of polyethylene combustion in inert or catalytic (Fe_2_O_3_) fluidised beds.
The data may be also used to the development of modelling of RDF combustion in fluidised bed.
Data from a real object will be of interest to engineers and scientists involved in modelling, among others from, Berkeley University of California [2], Politecnico di Milano [3], Lawrence Livermore National Laboratory [4], University of Southern California [5].

## 2 Data description

Correct modeling requires data from real objects. Ekpe Moses [6] used the data presented in the works [7,8] and created a simulation model of electricity production from the High Density Polyethylene (HDPE) waste. Thanks to that a theoretical framework for a waste plastic power plant using HDPE as the feed was proposed even though data from a real-life waste plastic power plant were not available. Shi et al. [9] undertook the numerical simulation of HDPE combustion because the fire risk of non-charring polymers is a big problem in civil engineering. That numerical results were validated by cone calorimeter experiments under spontaneous ignition condition. Fluidised bed combustion of polyethylene is interesting due to the use of Refuse-Derived Fuel (RDF) to generate energy.

**Table 1:** Parameters of fluidised bed reactor, bed material, and fluidising medium.

	Parameter, units	Magnitude	
Fluidised bed reactor	Shape	tube	
	Wall material	quartz	
	Inner diameter, mm	96	
	Height, mm	500	
	Heating	electrical	
	Sieve bottom	CrNi steel plate (1 mm thick), evenly distributed holes (diam. 0.6 mm; 6.25 cm^−2^)	
Types of fluidised bed	Type	inert	catalytic
	Material	cenospheres	cenospheres-Fe_2_O_3_
	Shape	spherical	
	Grain diameter, mm	0.25-0.30	
	Mass, g	300	
	Density, g/cm^3^	845	930
	Temperature,°C	500, 600, 700, 800, 900,	
Fluidising medium	Physical state	Gas	
	Composition	Air (79% N_2_, 21%O_2_)	
	Temperature,°C	25	
	Pressure, hPa	1013	
	Flow Rate, L/min @STP	30

**Table 2:** Composition of flue gases during PE combustion in a fluidised bed of cenospheres at 500 °C.

Temp.	Polymer	Mass	Time	CO2	CO	CH4	C2H6	C3H8	C2H4	C4+	HCHO	CH3CHO	Acrolein	C2H2	C6H6	CH3OH	CH3COOH	CH3COCH3
°C	-	mg	s	%_vol._	ppm													
500	PE	200	0	0.00	0.00	0.00	0.00	0.00	0.00	0.00	0.00	0.00	0.00	0.00	0.00	0.00	0.00	0.00
			7	0.15	299.81	5.21	0.00	0.00	16.20	20.01	41.53	22.26	0.00	2.42	5.97	2.36	0.00	0.00
			14	0.56	1503.31	26.51	0.00	0.00	96.20	181.82	191.69	179.45	0.00	5.65	44.75	1.92	0.61	0.00
			21	1.10	4159.61	106.34	0.00	0.00	349.98	464.50	529.10	973.21	0.00	10.96	198.98	33.00	10.58	0.00
			28	1.38	5106.00	178.11	0.00	0.00	387.75	702.56	546.02	1339.96	0.00	67.67	295.87	81.93	23.57	0.00
			35	0.56	2223.65	106.58	0.00	0.00	144.32	479.21	173.43	431.69	0.00	33.22	115.73	32.83	7.25	0.00
			42	0.45	867.60	64.65	0.00	0.00	12.15	212.36	41.37	173.76	0.00	10.88	32.16	19.64	2.14	0.00
			49	0.26	405.76	40.14	0.00	0.00	6.66	116.47	5.41	88.83	0.00	7.31	18.71	8.87	0.47	0.00
			56	0.15	226.25	27.38	0.00	0.00	0.00	77.23	0.00	69.84	0.00	4.50	13.28	6.99	0.00	0.00
			63	0.06	147.67	25.43	0.00	0.00	0.00	58.50	0.00	52.58	0.00	0.20	19.53	5.21	0.00	0.00
			70	0.06	117.79	21.74	0.00	0.00	0.00	47.06	0.00	0.00	0.00	0.00	12.65	4.07	0.00	0.00
			77	0.06	87.01	15.18	0.00	0.00	0.00	39.26	0.00	0.00	0.00	0.00	7.96	6.58	0.00	0.00
			84	0.04	78.34	15.74	0.00	0.00	0.00	34.15	0.00	0.00	0.00	0.00	8.33	2.23	0.00	0.00
			91	0.04	75.03	10.66	0.00	0.00	0.00	29.11	0.00	0.00	0.00	0.00	6.58	0.00	0.00	0.00
			98	0.00	60.67	10.90	0.00	0.00	0.00	26.52	0.00	0.00	0.00	0.00	8.95	0.00	0.00	0.00

		197	0	0.00	0.00	0.00	0.00	0.00	0.00	0.00	0.00	0.00	0.00	0.00	0.00	0.00	0.00	0.00
			7	0.15	351.11	7.59	0.00	0.00	16.13	26.23	42.16	21.11	0.00	5.81	0.52	0.00	0.00	0.00
			14	0.39	1599.31	18.28	0.00	0.00	109.52	213.88	200.36	220.19	0.00	16.35	48.36	8.36	1.15	3.74
			21	1.02	3697.30	84.47	0.00	0.00	308.08	505.09	466.05	810.13	0.00	50.06	132.63	47.66	14.86	16.28
			28	1.28	4112.86	158.23	0.00	0.00	291.21	548.47	436.01	777.85	0.00	56.35	169.94	54.24	16.02	8.88
			35	0.48	2031.36	78.56	0.00	0.00	119.23	418.40	156.34	354.70	0.00	31.04	94.26	49.19	4.44	2.26
			42	0.43	756.50	54.46	0.00	0.00	10.42	184.89	35.98	150.70	0.00	11.04	24.77	18.32	1.52	0.00
			49	0.20	312.84	30.76	0.00	0.00	0.00	96.74	3.59	131.61	0.00	7.44	22.74	4.98	0.00	0.00
			56	0.09	128.76	21.59	0.00	0.00	0.00	62.05	0.00	53.78	0.00	4.14	15.06	7.29	0.00	0.00
			63	0.06	70.58	19.91	0.00	0.00	0.00	43.40	0.00	34.28	0.00	3.57	11.36	4.63	0.00	0.00
			70	0.02	54.70	11.67	0.00	0.00	0.00	32.52	0.00	30.78	0.00	0.00	6.73	3.81	0.00	0.00
			77	0.00	45.23	9.99	0.00	0.00	0.00	25.16	0.00	19.00	0.00	0.00	9.93	4.22	0.00	0.00
			84	0.00	21.00	6.22	0.00	0.00	0.00	19.20	0.00	39.07	0.00	0.00	6.40	4.14	0.00	0.00

		300	0	0.00	0.00	0.00	0.00	0.00	0.00	0.00	0.00	0.00	0.00	0.00	0.00	0.00	0.00	0.00
			7	0.13	215.76	0.00	0.00	0.00	8.59	11.13	25.57	0.00	0.00	2.11	5.42	0.00	0.00	0.00
			14	0.55	1957.74	14.16	0.00	0.00	118.31	215.84	242.52	259.88	0.00	22.08	32.76	44.94	0.00	0.00
			21	1.14	5569.89	143.13	0.00	0.00	364.88	559.80	634.13	1234.37	0.00	28.03	261.33	70.76	17.78	0.00
			28	1.18	8422.66	230.20	0.00	0.00	558.54	1522.36	860.99	1976.82	0.00	41.33	639.24	100.32	36.08	0.00
			35	1.10	4719.66	154.66	0.00	0.00	329.78	743.30	475.38	1349.27	0.00	73.66	273.32	112.08	25.71	0.00
			42	0.40	1606.66	100.08	0.00	0.00	51.11	470.46	129.11	433.03	0.00	24.76	100.51	50.92	9.39	0.00
			49	0.36	656.75	75.48	0.00	0.00	6.75	222.70	22.44	187.59	0.00	13.93	45.35	16.68	3.72	0.00
			56	0.25	331.32	39.94	0.00	0.00	0.00	133.59	2.81	185.31	0.00	4.41	14.46	11.99	3.71	0.00
			63	0.10	172.71	28.06	0.00	0.00	0.00	91.03	0.00	82.90	0.00	2.36	9.27	9.61	2.07	0.00
			70	0.08	125.04	20.54	0.00	0.00	0.00	67.37	0.00	59.57	0.00	1.96	6.00	7.22	1.69	0.00
			77	0.04	98.06	10.47	0.00	0.00	0.70	52.27	0.00	31.05	0.00	2.36	11.40	3.33	0.00	0.00
			84	0.04	78.76	11.57	0.00	0.00	0.00	42.92	0.00	35.17	0.00	2.39	11.08	3.34	0.00	0.00
91	0.02		46.63	8.06	0.00	0.00	0.74	32.47	0.00	0.00	0.00	2.66	4.75	6.44	0.00	0.00
98	0.00		27.66	5.76	0.00	0.00	0.00	25.67	0.00	0.00	0.00	0.00	5.26	1.84	0.72	0.00

**Table 3:** Composition of flue gases during LLDPE combustion in a fluidised bed of cenospheres at 500 °C.

Temp.	Polymer	Mass	Time	CO2	CO	CH4	C2H6	C3H8	C2H4	C4+	HCHO	CH3CHO	Acrolein	C2H2	C6H6	CH3OH	CH3COOH	CH3COCH3
°C	-	mg	s	%_vol._	ppm													
500	LLDPE	206	0	0.00	0.00	0.00	0.00	0.00	0.00	0.00	0.00	0.00	0.00	0.00	0.00	0.00	0.00	0.00
			7	0.00	22.39	1.01	0.00	0.00	0.29	0.31	0.00	0.00	0.00	0.00	3.35	2.08	0.00	0.00
			14	0.14	238.79	6.23	0.00	0.00	8.17	13.36	27.52	0.00	0.00	1.50	1.63	4.52	0.00	0.00
			21	0.43	1016.62	6.26	0.00	0.00	57.72	115.17	116.50	79.46	0.00	10.94	27.80	14.58	0.00	0.00
			28	0.34	2569.08	37.70	0.00	0.00	184.32	394.00	319.21	455.30	0.00	26.52	62.83	21.28	5.18	0.00
			35	1.05	4558.95	83.39	0.00	0.00	307.45	532.24	529.00	1182.26	0.00	24.29	241.45	62.54	15.19	0.00
			42	1.17	3451.08	138.01	0.00	0.00	227.32	493.98	348.76	716.23	0.00	51.07	114.48	51.81	13.42	0.00
			49	0.57	1314.49	83.76	0.00	0.00	37.77	298.37	90.16	253.27	0.00	16.31	65.69	16.89	4.73	0.00
			56	0.33	578.75	40.20	0.00	0.00	12.52	141.04	20.61	109.72	0.00	5.50	20.30	10.70	1.55	0.00
			63	0.19	307.00	25.46	0.00	0.00	2.17	82.11	2.43	60.23	0.00	4.04	12.66	8.24	1.91	0.00
			70	0.10	162.64	19.04	0.00	0.00	0.00	57.24	0.00	50.69	0.00	0.67	17.41	6.35	0.00	0.00
			77	0.05	116.46	14.67	0.00	0.00	0.00	42.76	0.00	38.37	0.00	1.17	13.35	4.61	0.00	0.00
			84	0.03	83.61	16.46	0.00	0.00	0.00	33.32	0.00	28.84	0.00	0.00	15.03	5.02	0.00	0.00
			91	0.02	73.08	8.79	0.00	0.00	1.62	26.02	0.00	19.81	0.00	0.00	8.29	6.69	0.00	0.00
			98	0.03	75.16	0.00	0.00	0.00	0.00	21.04	0.00	15.86	0.00	0.00	11.61	3.10	0.00	0.00

		200	0	0.00	0.00	0.00	0.00	0.00	0.00	0.00	0.00	0.00	0.00	0.00	0.00	0.00	0.00	0.00
			7	0.13	234.29	1.84	0.00	0.00	10.79	12.16	25.59	3.20	0.00	0.15	1.62	0.00	0.00	0.00
			14	0.48	1081.39	0.00	0.00	0.00	61.32	113.27	123.24	69.97	0.00	8.69	25.74	0.00	0.00	0.00
			21	0.43	2354.43	30.41	0.00	0.00	165.80	389.63	285.79	390.02	0.00	27.17	70.77	12.31	3.11	0.00
			28	1.06	3492.08	70.08	0.00	0.00	269.87	516.71	434.33	764.87	0.00	43.10	117.09	43.13	12.74	0.00
			35	0.48	2144.43	55.58	0.00	0.00	136.25	431.07	203.71	384.11	0.00	32.59	83.07	24.03	6.03	0.00
			42	0.60	1218.88	45.88	0.00	0.00	38.52	238.84	83.09	191.15	0.00	6.05	72.48	0.00	1.71	0.00
			49	0.47	823.68	36.59	0.00	0.00	17.17	130.74	41.61	86.77	0.00	8.84	18.90	0.00	0.00	0.00
			56	0.28	415.30	22.36	0.00	0.00	10.84	72.13	16.65	43.41	0.00	5.78	8.48	0.00	0.00	0.00
			63	0.19	230.99	20.39	0.00	0.00	0.00	44.79	6.77	67.11	0.00	1.25	0.00	0.00	0.00	0.00
			70	0.11	143.09	10.36	0.00	0.00	0.00	29.65	1.38	26.30	0.00	0.78	0.00	0.00	0.00	0.00
			77	0.07	92.13	10.94	0.00	0.00	0.00	20.72	0.00	13.62	0.00	0.00	0.00	0.00	0.00	0.00
			84	0.06	48.99	8.48	0.00	0.00	2.38	12.56	0.00	0.00	0.00	0.00	0.00	0.00	0.00	0.00
			91	0.02	21.09	3.65	0.00	0.00	0.00	7.50	0.00	4.85	0.00	0.00	0.00	0.00	0.00	0.00
			98	0.02	0.00	0.80	0.00	0.00	0.00	4.82	0.00	3.44	0.00	0.00	0.00	0.00	0.00	0.00

		304	0	0.00	0.00	0.00	0.00	0.00	0.00	0.00	0.00	0.00	0.00	0.00	0.00	0.00	0.00	0.00
			7	0.04	48.62	0.91	0.00	0.00	4.64	3.26	7.95	0.00	0.00	0.76	1.98	1.42	0.00	0.00
			14	0.45	1052.92	0.00	0.00	0.00	57.00	104.00	123.71	65.80	0.00	12.98	25.30	0.45	0.00	0.00
			21	0.39	2574.57	29.82	0.00	0.00	183.43	392.21	308.81	416.27	0.00	29.57	66.39	18.86	4.64	0.00
			28	1.18	4693.52	81.81	0.00	0.00	338.28	573.17	565.86	1079.77	0.00	28.75	255.56	60.76	14.45	0.00
			35	1.46	8270.10	191.61	0.00	0.00	532.98	1300.75	813.13	1854.74	0.00	94.28	698.19	78.55	33.78	0.00
			42	1.18	3811.63	156.64	0.00	0.00	261.24	614.26	373.04	800.70	0.00	49.59	150.04	75.83	17.19	0.00
			49	0.54	1361.72	78.56	0.00	0.00	36.18	396.50	92.60	335.70	0.00	21.94	81.48	23.22	7.45	0.00
			56	0.31	588.96	58.18	0.00	0.00	18.74	197.32	17.42	149.26	0.00	9.29	21.01	18.92	3.57	0.00
			63	0.14	280.72	37.29	0.00	0.00	5.12	119.85	0.00	194.74	0.00	5.45	11.52	16.76	3.53	0.00
			70	0.08	141.87	35.73	0.00	0.00	4.91	81.31	0.00	138.09	0.00	4.02	19.56	14.90	2.37	0.00
			77	0.08	118.05	20.45	0.00	0.00	0.00	62.66	0.00	57.91	0.00	3.28	3.67	7.16	1.18	0.00
			84	0.06	112.77	15.15	0.00	0.00	1.51	49.28	0.00	33.56	0.00	0.49	11.21	7.88	0.00	0.00
91	0.06		93.31	12.40	0.00	0.00	0.00	40.08	0.00	35.29	0.00	0.31	9.00	4.87	0.00	0.00
98	0.04		66.52	7.99	0.00	0.00	0.00	31.89	0.00	29.37	0.00	1.03	12.42	4.33	0.00	0.00

**Table 4:** Composition of flue gases during HDPE combustion in a fluidised bed of cenospheres at 500 °C.

Temp.	Polymer	Mass	Time	CO2	CO	CH4	C2H6	C3H8	C2H4	C4+	HCHO	CH3CHO	Acrolein	C2H2	C6H6	CH3OH	CH3COOH	CH3COCH3
°C	-	mg	s	%_vol._	ppm													
500	HDPE	199	0	0.00	0.00	0.00	0.00	0.00	0.00	0.00	0.00	0.00	0.00	0.00	0.00	0.00	0.00	0.00
			7	0.08	201.67	0.00	0.00	0.00	8.11	11.52	20.57	0.00	0.00	0.00	1.89	0.00	0.00	0.00
			14	0.33	750.69	0.00	0.00	0.00	29.88	64.89	81.91	44.70	0.00	6.64	23.62	0.00	0.00	0.00
			21	0.54	1315.66	9.36	0.00	0.00	80.11	165.86	139.54	136.53	0.00	16.08	49.89	0.35	0.00	0.00
			28	0.48	2033.35	26.61	0.00	0.00	141.78	304.17	222.47	288.69	0.00	23.76	67.49	12.16	0.00	0.00
			35	0.39	3272.18	48.96	0.00	0.00	234.71	517.10	358.57	563.12	0.00	43.75	116.03	29.82	7.66	0.00
			42	0.37	3094.39	60.24	0.00	0.00	217.38	487.05	329.66	553.68	0.00	37.81	114.51	31.65	7.84	0.00
			49	0.46	2123.33	49.56	0.00	0.00	124.39	390.61	181.67	348.52	0.00	30.07	88.01	21.85	3.44	0.00
			56	0.42	851.79	42.99	0.00	0.00	12.72	184.91	48.00	151.08	0.00	10.81	49.60	18.22	0.23	0.00
			63	0.19	371.66	32.60	0.00	0.00	3.17	95.15	11.16	67.28	0.00	5.11	11.44	5.62	0.00	0.00
			70	0.12	212.28	30.55	0.00	0.00	1.20	59.41	0.00	41.92	0.00	3.67	16.93	3.55	0.00	0.00
			77	0.06	128.71	16.17	0.00	0.00	2.59	41.09	0.00	24.94	0.00	2.14	12.83	4.42	0.00	0.00
			84	0.08	76.64	13.03	0.00	0.00	0.00	28.95	0.00	21.03	0.00	3.13	1.49	3.32	0.00	0.00
			91	0.00	58.54	10.89	0.00	0.00	0.00	22.85	0.00	19.86	0.00	0.00	0.00	2.03	0.00	0.00

		201	0	0.00	0.00	0.00	0.00	0.00	0.00	0.00	0.00	0.00	0.00	0.00	0.00	0.00	0.00	0.00
			7	0.03	60.89	0.00	0.00	0.00	0.00	2.20	5.75	0.06	0.00	0.63	0.28	0.00	0.00	0.00
			14	0.29	567.00	12.70	0.00	0.00	18.62	40.65	59.99	18.42	0.00	7.62	5.08	0.00	0.00	0.00
			21	0.32	1448.56	7.89	0.00	0.00	94.55	186.98	161.92	185.39	0.00	15.36	47.63	6.69	0.00	0.00
			28	0.24	2423.43	28.63	0.00	0.00	163.10	362.26	273.27	401.79	0.00	26.78	78.37	17.76	5.46	0.00
			35	1.09	3714.69	37.35	0.00	0.00	250.09	403.08	398.11	778.27	0.00	57.93	130.26	40.27	12.00	0.00
			42	1.07	2861.33	118.42	0.00	0.00	179.48	366.33	258.75	536.75	0.00	43.91	101.70	31.04	7.80	0.00
			49	0.47	1546.37	67.24	0.00	0.00	48.23	304.21	112.88	253.86	0.00	24.98	69.63	32.94	3.35	0.00
			56	0.34	641.99	36.88	0.00	0.00	24.86	143.45	29.24	117.24	0.00	8.26	29.97	11.93	1.84	0.00
			63	0.17	254.82	24.11	0.00	0.00	0.00	76.87	2.77	120.49	0.00	1.58	6.95	4.85	0.24	0.00
			70	0.06	120.67	14.61	0.00	0.00	0.00	46.16	0.00	85.94	0.00	0.00	10.57	5.71	0.00	0.00
			77	0.03	74.74	10.88	0.00	0.00	0.26	34.10	0.00	21.99	0.00	0.00	7.11	3.78	0.00	0.00
			84	0.02	49.19	12.53	0.00	0.00	0.00	25.06	0.00	19.54	0.00	0.00	4.47	2.78	0.00	0.00
			91	0.00	24.31	4.47	0.00	0.00	0.00	17.64	0.00	18.56	0.00	0.00	1.99	2.89	0.00	0.00
			98	0.00	7.37	3.99	0.00	0.00	0.00	12.15	0.00	10.24	0.00	0.00	0.00	3.17	0.00	0.00

		307	0	0.00	0.00	0.00	0.00	0.00	0.00	0.00	0.00	0.00	0.00	0.00	0.00	0.00	0.00	0.00
			7	0.17	246.58	3.04	0.00	0.00	8.78	14.89	30.82	0.00	0.00	0.18	2.19	0.00	0.00	0.00
			14	0.42	1709.56	7.42	0.00	0.00	97.85	191.47	194.19	200.65	0.00	15.25	48.32	4.38	0.00	0.00
			21	1.16	4812.37	63.28	0.00	0.00	334.51	453.78	557.32	1019.53	0.00	54.98	150.97	50.16	16.50	0.00
			28	1.40	7286.64	167.46	0.00	0.00	443.92	1007.47	703.84	1479.58	0.00	38.46	296.20	113.62	25.72	0.00
			35	1.29	4893.61	120.89	0.00	0.00	301.50	629.08	446.64	898.60	0.00	70.03	232.41	73.10	19.10	0.00
			42	0.52	2788.83	151.21	0.00	0.00	155.19	457.74	211.60	517.68	0.00	40.42	102.47	41.77	10.01	0.00
			49	0.63	1338.54	69.46	0.00	0.00	28.37	299.44	65.89	258.17	0.00	17.28	77.04	28.32	4.75	0.00
			56	0.33	570.01	52.86	0.00	0.00	13.47	159.77	13.40	130.70	0.00	9.02	19.19	12.68	2.08	0.00
			63	0.18	311.27	35.24	0.00	0.00	4.80	99.36	0.00	85.58	0.00	3.05	7.95	12.44	2.91	0.00
			70	0.13	201.02	34.06	0.00	0.00	0.00	70.58	0.00	65.34	0.00	2.28	17.55	7.97	1.71	0.00
			77	0.06	123.30	18.84	0.00	0.00	0.00	54.43	0.00	104.77	0.00	4.18	11.57	5.65	1.51	0.00
			84	0.07	100.43	12.49	0.00	0.00	0.61	41.31	0.00	34.67	0.00	1.99	9.02	4.45	1.45	0.00
91	0.04		73.95	13.58	0.00	0.00	0.18	32.60	0.00	24.07	0.00	2.83	7.51	5.06	0.53	0.00
98	0.02		58.88	11.85	0.00	0.00	0.00	26.20	0.00	27.86	0.00	1.20	4.95	2.96	0.00	0.00

**Table 5:** Composition of flue gases during PE combustion in a fluidised bed of cenospheres at 600 °C.

Temp.	Polymer	Mass	Time	CO2	CO	CH4	C2H6	C3H8	C2H4	C4+	HCHO	CH3CHO	Acrolein	C2H2	C6H6	CH3OH	CH3COOH	CH3COCH3
°C	-	mg	s	%_vol._	ppm													
600	PE	203	0	0.00	0.00	0.00	0.00	0.00	0.00	0.00	0.00	0.00	0.00	0.00	0.00	0.00	0.00	0.00
			7	0.05	133.37	5.37	4.03	0.00	21.05	10.42	18.71	4.86	0.18	4.51	5.54	0.83	0.00	0.00
			14	0.88	2202.83	99.79	27.79	0.00	424.42	143.86	221.25	149.72	21.26	44.33	116.67	41.01	3.31	0.00
			21	3.33	2886.33	135.31	51.02	0.00	511.54	165.02	219.72	164.98	15.08	39.32	136.59	44.72	3.54	0.00
			28	1.28	2579.33	98.45	22.67	0.00	409.90	135.68	164.95	108.50	11.97	36.59	121.58	34.23	5.81	0.00
			35	0.69	1014.78	27.65	0.00	0.00	128.30	51.99	44.62	24.13	0.00	6.37	38.76	0.00	2.71	0.00
			42	0.30	409.39	14.34	0.00	0.00	28.51	19.32	12.21	0.00	0.00	0.00	11.39	0.00	0.00	0.00
			49	0.16	162.78	6.49	0.00	0.00	4.40	9.73	2.24	0.00	0.00	0.00	3.63	0.56	0.00	0.00
			56	0.09	73.23	2.62	0.00	0.00	0.00	5.90	2.57	0.00	0.00	0.00	3.06	0.00	0.00	0.00
			63	0.07	30.78	1.58	0.00	0.00	0.77	4.38	0.86	0.00	0.00	0.00	1.37	0.00	0.00	0.00

		199	0	0.00	0.00	0.00	0.00	0.00	0.00	0.00	0.00	0.00	0.00	0.00	0.00	0.00	0.00	0.00
			7	0.19	386.35	21.24	19.20	0.00	88.80	27.52	50.41	25.01	8.58	8.89	22.77	5.73	0.00	0.00
			14	1.32	2494.17	110.06	27.86	0.00	438.22	118.59	210.75	154.08	27.38	34.40	114.88	20.69	3.84	0.00
			21	3.18	3441.70	149.86	35.28	0.00	562.18	133.85	239.30	179.74	20.11	41.59	145.24	44.89	5.09	0.00
			28	1.05	1951.67	64.50	15.22	0.00	284.09	81.56	113.80	62.54	9.80	23.13	74.82	11.35	3.01	0.00
			35	0.43	693.19	24.37	11.11	0.00	77.54	28.17	34.50	5.06	0.00	5.04	21.31	0.00	1.63	0.00
			42	0.21	261.46	8.45	1.38	0.00	17.48	11.47	10.30	0.57	1.87	1.05	7.29	0.00	0.10	0.00
			49	0.09	104.09	2.60	0.00	0.00	5.35	6.11	2.61	0.00	1.70	0.00	3.49	0.00	0.00	0.00
			56	0.05	47.94	2.18	0.00	0.00	1.34	3.49	0.91	0.22	0.00	0.00	1.96	0.00	0.00	0.00
			63	0.00	18.61	0.72	0.00	0.00	1.17	2.80	0.03	0.00	0.00	0.00	0.00	0.00	0.00	0.00

		102	0	0.00	0.00	0.00	0.00	0.00	0.00	0.00	0.00	0.00	0.00	0.00	0.00	0.00	0.00	0.00
			7	0.00	8.32	0.00	0.99	0.00	0.00	0.11	0.00	0.00	2.16	0.00	0.00	0.00	0.00	0.00
			14	0.34	866.48	42.60	20.36	0.00	197.47	61.23	103.16	64.44	16.02	18.16	55.74	18.40	0.98	0.00
			21	1.11	3144.08	154.31	34.19	0.00	578.55	186.76	270.92	203.43	9.54	59.70	169.11	59.94	6.43	0.00
			28	0.40	2143.66	106.16	15.99	0.00	412.61	138.34	178.61	118.14	2.02	34.95	123.17	40.31	5.20	0.00
			35	0.33	676.47	29.37	11.77	0.00	127.97	49.93	45.06	25.86	0.00	3.21	34.85	9.39	0.36	0.00
			42	0.16	216.64	13.96	6.49	0.00	26.23	18.87	11.06	2.03	0.00	0.67	9.22	0.00	0.00	0.00
			49	0.06	65.46	5.35	0.00	0.00	0.23	8.65	1.29	4.17	0.00	0.00	5.52	0.00	0.00	0.00
56	0.04		21.14	1.75	0.00	0.00	0.00	4.91	0.00	1.79	0.00	0.10	1.99	0.00	0.00	0.00
63	0.01		10.10	0.71	0.00	0.00	0.00	3.25	0.00	0.00	0.00	0.00	0.20	0.00	0.00	0.00

**Table 6:** Composition of flue gases during LLDPE combustion in a fluidised bed of cenospheres at 600 °C.

Temp.	Polymer	Mass	Time	CO2	CO	CH4	C2H6	C3H8	C2H4	C4+	HCHO	CH3CHO	Acrolein	C2H2	C6H6	CH3OH	CH3COOH	CH3COCH3
°C	-	mg	s	%_vol._	ppm													
600	LLDPE	196	0	0.00	0.00	0.00	0.00	0.00	0.00	0.00	0.00	0.00	0.00	0.00	0.00	0.00	0.00	0.00
			7	0.12	228.77	12.66	12.53	0.00	50.55	17.36	31.17	11.75	2.88	6.55	11.96	0.87	0.00	0.00
			14	0.93	2562.47	109.83	33.57	0.00	474.49	128.16	230.71	163.76	23.42	40.80	132.73	38.34	2.26	0.00
			21	3.22	3307.06	144.34	43.25	0.00	522.96	113.83	208.19	143.91	20.42	44.91	135.17	35.05	4.90	0.00
			28	1.50	2056.90	81.40	24.42	0.00	324.41	68.71	105.23	62.62	7.58	25.45	77.18	7.32	3.44	0.00
			35	0.69	747.08	29.51	12.16	0.00	87.02	23.48	27.55	9.29	0.00	5.14	20.43	0.00	0.05	0.00
			42	0.32	269.17	7.62	6.23	0.00	19.30	8.88	8.13	0.18	0.00	0.00	6.46	0.00	0.00	0.00
			49	0.12	97.94	4.24	0.62	0.00	6.68	4.13	1.30	1.73	0.00	0.00	1.51	0.00	0.00	0.00
			56	0.04	32.65	1.37	0.00	0.00	0.00	2.19	0.00	0.66	0.00	1.87	0.43	0.00	0.00	0.00
			63	0.05	10.70	0.73	0.00	0.00	0.82	1.26	0.00	1.57	0.00	2.12	0.50	0.00	0.00	0.00

		204	0	0.00	0.00	0.00	0.00	0.00	0.00	0.00	0.00	0.00	0.00	0.00	0.00	0.00	0.00	0.00
			7	0.00	8.49	0.00	0.00	0.00	3.04	0.42	0.80	0.00	0.00	0.80	0.56	0.70	0.00	0.00
			14	0.37	1254.96	53.51	23.77	0.00	252.91	57.83	140.96	84.66	38.55	17.43	66.61	25.21	0.00	0.00
			21	2.12	4116.47	173.50	54.76	0.00	645.08	137.39	329.10	264.34	27.94	52.40	156.75	59.50	10.73	0.00
			28	2.79	2442.30	89.25	25.05	0.00	360.25	69.42	146.14	100.39	25.40	28.72	86.58	22.17	4.73	0.00
			35	1.23	1207.27	38.52	8.85	0.00	169.43	34.16	51.43	36.93	0.00	13.28	35.12	0.00	0.53	0.00
			42	0.48	474.61	13.20	7.94	0.00	40.72	12.04	14.29	7.55	0.00	3.37	9.72	0.67	0.00	0.00
			49	0.16	171.12	4.26	0.00	0.00	12.09	5.04	1.09	0.39	0.00	1.78	3.84	0.00	0.00	0.00
			56	0.09	69.08	2.67	0.00	0.00	5.93	3.09	2.36	0.00	0.00	2.34	2.67	0.00	0.00	0.00
			63	0.03	24.73	0.19	0.00	0.00	0.00	1.72	1.89	0.00	0.00	0.55	1.23	0.00	0.00	0.00

		100	0	0.00	0.00	0.00	0.00	0.00	0.00	0.00	0.00	0.00	0.00	0.00	0.00	0.00	0.00	0.00
			7	0.06	59.19	1.29	0.00	0.00	17.49	3.83	6.47	2.42	0.00	0.30	2.63	0.00	0.00	0.00
			14	0.29	1404.74	68.76	30.92	0.00	321.59	89.63	151.85	93.66	24.06	19.86	83.99	26.82	0.00	0.00
			21	1.11	3010.35	147.83	45.47	0.00	573.36	158.88	258.26	198.30	11.58	52.92	165.38	50.78	5.21	0.00
			28	0.51	2418.60	109.79	61.55	0.00	443.18	102.41	178.79	130.64	12.09	33.11	108.74	17.58	6.35	0.00
			35	0.32	723.56	30.09	5.79	0.00	134.97	34.74	50.28	30.36	0.00	3.17	30.89	0.00	2.82	0.00
			42	0.15	219.77	11.12	0.15	0.00	35.37	12.54	12.63	8.22	0.00	0.00	8.61	0.00	0.00	0.00
			49	0.06	66.15	4.55	0.00	0.00	9.42	5.81	2.48	0.96	0.00	0.00	4.20	0.50	0.00	0.00
56	0.03		26.00	0.71	0.00	0.00	6.30	3.65	1.89	1.26	0.00	0.00	2.48	1.76	0.00	0.00
63	0.03		10.02	0.00	0.00	0.00	5.40	2.24	1.61	3.38	0.00	0.00	0.30	0.00	0.00	0.00

**Table 7:** Composition of flue gases during HDPE combustion in a fluidised bed of cenospheres at 600 °C.

Temp.	Polymer	Mass	Time	CO2	CO	CH4	C2H6	C3H8	C2H4	C4+	HCHO	CH3CHO	Acrolein	C2H2	C6H6	CH3OH	CH3COOH	CH3COCH3
°C	-	mg	s	%_vol._	ppm													
600	HDPE	200	0	0.00	0.00	0.00	0.00	0.00	0.00	0.00	0.00	0.00	0.00	0.00	0.00	0.00	0.00	0.00
			7	0.09	68.80	4.77	4.43	0.00	15.17	6.46	8.69	2.50	4.05	2.88	2.57	0.00	0.00	0.00
			14	1.19	2423.92	117.81	35.14	0.00	496.98	133.99	225.52	159.32	21.35	47.09	127.21	43.90	0.00	0.00
			21	3.53	3704.89	155.23	61.34	0.00	557.17	139.14	230.53	161.77	22.39	45.36	140.20	42.28	0.00	0.00
			28	1.30	2910.13	104.22	22.79	0.00	424.80	103.50	165.17	108.92	9.11	36.65	110.45	29.54	0.00	0.00
			35	0.61	961.18	27.95	2.39	0.00	126.81	37.62	44.05	10.67	4.18	10.31	31.32	6.32	0.00	0.00
			42	0.31	338.71	11.99	0.00	0.00	30.29	13.25	11.12	0.00	0.11	2.30	8.17	0.00	0.00	0.00
			49	0.13	131.24	4.37	0.00	0.00	5.36	6.07	2.68	0.00	0.00	1.39	3.53	0.00	0.00	0.00
			56	0.09	48.37	0.77	0.00	0.00	1.82	3.87	0.00	0.00	0.00	0.74	1.60	0.00	0.00	0.00
			63	0.02	15.97	0.00	0.00	0.00	0.00	2.66	0.00	0.00	0.00	0.00	0.00	0.00	0.00	0.00

		298	0	0.00	0.00	0.00	0.00	0.00	0.00	0.00	0.00	0.00	0.00	0.00	0.00	0.00	0.00	0.00
			7	0.18	244.72	12.73	12.09	0.00	63.74	19.72	35.49	14.61	2.07	6.13	15.75	5.34	0.00	0.00
			14	1.24	2190.19	91.52	22.18	0.00	400.40	151.10	208.43	140.70	7.55	40.66	122.34	40.75	0.00	0.00
			21	5.04	2603.38	86.59	11.27	0.00	342.43	82.77	136.67	81.08	13.68	32.38	139.26	22.14	0.00	0.00
			28	3.01	3766.58	137.37	70.24	0.00	515.85	90.73	188.73	116.75	25.08	42.28	126.54	15.71	0.00	0.00
			35	1.09	1515.04	43.89	14.71	0.00	192.55	35.20	59.87	32.15	2.66	13.24	42.13	4.64	0.00	0.00
			42	0.50	530.02	13.54	7.01	0.00	49.47	11.97	15.10	3.54	0.28	4.06	10.79	0.00	0.00	0.00
			49	0.20	185.50	4.79	0.22	0.00	13.08	5.44	3.46	0.00	0.00	0.00	5.24	0.00	0.00	0.00
			56	0.07	75.70	1.45	0.00	0.00	6.41	3.11	0.94	0.00	0.00	0.00	2.47	0.00	0.00	0.00
			63	0.03	27.35	1.19	0.00	0.00	4.56	2.10	0.00	0.00	0.00	0.00	2.49	0.00	0.00	0.00

		100	0	0.00	0.00	0.00	0.00	0.00	0.00	0.00	0.00	0.00	0.00	0.00	0.00	0.00	0.00	0.00
			7	0.21	315.79	23.36	17.20	0.00	89.16	26.09	42.50	25.52	2.44	6.76	21.83	6.11	0.00	0.00
			14	1.65	2828.36	119.56	26.63	0.00	450.59	106.17	199.91	147.44	17.44	37.20	126.03	37.29	0.00	0.00
			21	0.67	2055.69	88.05	22.12	0.00	347.89	101.69	137.65	93.81	14.57	28.93	98.24	26.58	0.00	0.00
			28	0.38	651.33	25.60	7.55	0.00	109.23	36.44	35.95	20.70	0.00	2.23	29.81	0.00	0.00	0.00
			35	0.20	208.44	11.84	3.54	0.00	25.08	12.94	8.39	3.75	0.00	0.00	9.60	0.00	0.00	0.00
			42	0.07	70.94	5.47	1.78	0.00	5.88	6.05	2.39	0.45	0.00	0.00	3.72	0.00	0.00	0.00
			49	0.04	26.54	2.19	0.00	0.00	3.12	3.07	0.20	1.11	0.00	0.00	2.23	0.00	0.00	0.00
56	0.01		10.51	2.43	0.00	0.00	3.68	2.07	0.00	0.00	0.00	0.00	1.72	0.00	0.00	0.00
63	0.01		4.83	1.09	0.00	0.00	0.00	1.46	0.00	1.33	0.00	0.00	1.33	0.00	0.00	0.00

**Table 8:** Composition of flue gases during PE combustion in a fluidised bed of cenospheres at 700 °C.

Temp.	Polymer	Mass	Time	CO2	CO	CH4	C2H6	C3H8	C2H4	C4+	HCHO	CH3CHO	Acrolein	C2H2	C6H6	CH3OH	CH3COOH	CH3COCH3
°C	-	mg	s	%_vol._	ppm													
700	PE	200	0	0.00	0.00	0.00	0.00	0.00	0.00	0.00	0.00	0.00	0.00	0.00	0.00	0.00	0.00	0.00
			7	0.01	52.69	0.48	0.00	0.00	3.32	0.00	0.59	0.00	0.00	0.00	0.00	0.00	0.00	0.00
			14	2.22	755.96	3.18	0.00	0.00	9.27	0.00	1.09	0.00	0.00	0.00	0.00	0.00	0.00	0.00
			21	4.14	1500.08	8.05	0.00	0.00	4.30	0.00	1.72	0.00	0.00	0.00	0.00	0.00	0.00	0.00
			28	1.85	992.54	6.37	0.00	0.00	1.30	0.00	1.19	0.00	0.00	0.00	0.00	0.00	0.00	0.00
			35	0.59	269.06	1.55	0.00	0.00	0.00	0.00	0.35	0.00	0.00	0.00	0.00	0.00	0.00	0.00
			42	0.15	65.90	0.70	0.00	0.00	0.00	0.00	0.00	0.00	0.00	0.00	0.00	0.00	0.00	0.00
			49	0.01	17.65	0.24	0.00	0.00	0.07	0.00	0.00	0.00	0.00	0.00	0.00	0.00	0.00	0.00
			56	0.00	4.84	0.00	0.00	0.00	0.00	0.00	0.00	0.00	0.00	0.00	0.00	0.00	0.00	0.00
			63	0.00	1.23	0.00	0.00	0.00	0.00	0.00	0.00	0.00	0.00	0.00	0.00	0.00	0.00	0.00

		199	0	0.00	0.00	0.00	0.00	0.00	0.00	0.00	0.00	0.00	0.00	0.00	0.00	0.00	0.00	0.00
			7	1.25	693.12	4.16	0.00	0.00	6.39	0.00	1.19	0.00	0.00	0.00	0.00	0.00	0.00	0.00
			14	4.45	1115.55	5.49	0.00	0.00	4.44	0.00	1.68	0.00	0.00	0.00	0.00	0.00	0.00	0.00
			21	2.16	1035.92	9.37	0.00	0.00	4.54	0.00	1.29	0.00	0.00	0.00	0.00	0.00	0.00	0.00
			28	0.71	306.90	2.94	0.00	0.00	1.16	0.00	0.00	0.00	0.00	0.00	0.00	0.00	0.00	0.00
			35	0.26	76.59	0.53	0.00	0.00	0.00	0.00	0.00	0.00	0.00	0.00	0.00	0.00	0.00	0.00
			42	0.05	17.87	0.30	0.00	0.00	0.08	0.00	0.00	0.00	0.00	0.00	0.00	0.00	0.00	0.00
			49	0.01	2.93	0.00	0.00	0.00	0.00	0.00	0.00	0.00	0.00	0.00	0.00	0.00	0.00	0.00
			56	0.01	0.00	0.00	0.00	0.00	0.00	0.00	0.00	0.00	0.00	0.00	0.00	0.00	0.00	0.00
			63	0.00	0.00	0.12	0.00	0.00	0.09	0.00	0.00	0.00	0.00	0.00	0.00	0.00	0.00	0.00

		102	0	0.00	0.00	0.00	0.00	0.00	0.00	0.00	0.00	0.00	0.00	0.00	0.00	0.00	0.00	0.00
			7	0.11	121.76	1.94	0.00	0.00	0.00	0.00	0.64	0.00	0.00	0.00	0.00	0.00	0.00	0.00
			14	1.87	791.81	6.17	0.00	0.00	5.16	0.00	2.14	0.00	0.00	0.00	0.00	0.00	0.00	0.00
			21	1.64	1232.15	10.00	0.00	0.00	7.55	0.00	2.78	0.00	0.00	0.00	0.00	0.00	0.00	0.00
			28	0.58	388.63	4.65	0.00	0.00	0.00	0.00	0.77	0.00	0.00	0.00	0.00	0.00	0.00	0.00
			35	0.17	100.31	1.55	0.00	0.00	0.00	0.00	0.09	0.00	0.00	0.00	0.00	0.00	0.00	0.00
			42	0.04	25.89	0.41	0.00	0.00	0.00	0.00	0.00	0.00	0.00	0.00	0.00	0.00	0.00	0.00
			49	0.01	6.24	0.24	0.00	0.00	0.00	0.00	0.00	0.00	0.00	0.00	0.00	0.00	0.00	0.00
56	0.00		1.49	0.24	0.00	0.00	0.00	0.00	0.00	0.00	0.00	0.00	0.00	0.00	0.00	0.00
63	0.00		0.64	0.00	0.00	0.00	0.00	0.00	0.00	0.00	0.00	0.00	0.00	0.00	0.00	0.00

**Table 9:** Composition of flue gases during LLDPE combustion in a fluidised bed of cenospheres at 700 °C.

Temp.	Polymer	Mass	Time	CO2	CO	CH4	C2H6	C3H8	C2H4	C4+	HCHO	CH3CHO	Acrolein	C2H2	C6H6	CH3OH	CH3COOH	CH3COCH3
°C	-	mg	s	%_vol._	ppm													
700	LLDPE	204	0	0.00	0.00	0.00	0.00	0.00	0.00	0.00	0.00	0.00	0.00	0.00	0.00	0.00	0.00	0.00
			7	0.02	16.67	0.00	0.00	0.00	0.52	0.00	0.98	0.00	0.00	0.00	0.00	0.00	0.00	0.00
			14	2.03	815.74	6.65	0.00	0.00	8.36	0.00	3.81	0.00	0.00	0.00	0.00	0.00	0.00	0.00
			21	4.83	1435.82	9.14	0.00	0.00	7.94	0.00	3.07	0.00	0.00	0.00	0.00	0.00	0.00	0.00
			28	1.95	892.21	7.64	0.00	0.00	3.61	0.00	2.12	0.00	0.00	0.00	0.00	0.00	0.00	0.00
			35	0.65	237.66	2.09	0.00	0.00	0.00	0.00	0.52	0.00	0.00	0.00	0.00	0.00	0.00	0.00
			42	0.21	61.88	0.06	0.00	0.00	0.00	0.00	0.39	0.00	0.00	0.00	0.00	0.00	0.00	0.00
			49	0.10	15.53	0.00	0.00	0.00	0.00	0.00	0.34	0.00	0.00	0.00	0.00	0.00	0.00	0.00
			56	0.04	3.10	0.10	0.00	0.00	0.00	0.00	0.00	0.00	0.00	0.00	0.00	0.00	0.00	0.00
			63	0.02	1.35	0.00	0.00	0.00	0.00	0.00	0.00	0.00	0.00	0.00	0.00	0.00	0.00	0.00

		203	0	0.00	0.00	0.00	0.00	0.00	0.00	0.00	0.00	0.00	0.00	0.00	0.00	0.00	0.00	0.00
			7	0.01	4.15	1.42	0.00	0.00	0.47	0.00	0.49	0.00	0.00	0.00	0.00	0.00	0.00	0.00
			14	1.23	812.47	7.59	0.00	0.00	8.47	0.00	5.43	0.00	0.00	0.00	0.00	0.00	0.00	0.00
			21	4.48	2023.14	13.72	0.00	0.00	10.03	0.00	3.98	0.00	0.00	0.00	0.00	0.00	0.00	0.00
			28	2.47	1527.64	12.99	0.00	0.00	5.84	0.00	3.14	0.00	0.00	0.00	0.00	0.00	0.00	0.00
			35	0.83	410.94	5.44	0.00	0.00	0.11	0.00	3.27	0.00	0.00	0.00	0.00	0.00	0.00	0.00
			42	0.31	104.30	2.82	0.00	0.00	0.00	0.00	1.37	0.00	0.06	0.00	0.00	0.00	0.00	0.00
			49	0.07	27.92	1.24	0.00	0.00	0.00	0.00	1.45	0.00	0.00	0.00	0.00	0.00	0.00	0.00
			56	0.06	7.70	1.30	0.00	0.00	0.00	0.00	0.14	0.00	0.00	0.00	0.00	0.00	0.00	0.00
			63	0.03	2.13	1.51	0.00	0.00	0.00	0.00	0.08	0.00	0.00	0.00	0.00	0.00	0.00	0.00

		297	0	0.00	0.00	0.00	0.00	0.00	0.00	0.00	0.00	0.00	0.00	0.00	0.00	0.00	0.00	0.00
			7	0.68	563.95	6.67	0.00	0.00	10.89	0.00	0.81	0.00	0.00	0.00	0.00	0.00	0.00	0.00
			14	6.04	1037.57	10.92	0.00	0.00	11.07	0.00	2.80	0.00	0.00	0.00	0.00	0.00	0.00	0.00
			21	4.96	1611.66	14.81	0.00	0.00	7.43	0.00	2.96	0.00	0.00	0.00	0.00	0.00	0.00	0.00
			28	1.70	621.73	4.99	0.00	0.00	4.60	0.00	0.53	0.00	0.00	0.00	0.00	0.00	0.00	0.00
			35	0.56	164.01	1.47	0.00	0.00	3.40	0.00	0.25	0.00	0.00	0.00	0.00	0.00	0.00	0.00
			42	0.16	42.77	1.42	0.00	0.00	0.00	0.00	0.00	0.00	0.00	0.00	0.00	0.00	0.00	0.00
			49	0.01	11.11	0.00	0.00	0.00	0.00	0.00	0.00	0.00	0.00	0.00	0.00	0.00	0.06	0.00
56	0.01		3.12	0.00	0.00	0.00	0.00	0.00	0.00	0.00	0.00	0.00	0.00	0.00	0.00	0.00
63	0.03		1.42	0.00	0.00	0.00	0.00	0.00	0.00	0.00	0.00	0.00	0.00	0.00	0.00	0.00

**Table 10:** Composition of flue gases during HDPE combustion in a fluidised bed of cenospheres at 700 °C.

Temp.	Polymer	Mass	Time	CO2	CO	CH4	C2H6	C3H8	C2H4	C4+	HCHO	CH3CHO	Acrolein	C2H2	C6H6	CH3OH	CH3COOH	CH3COCH3
°C	-	mg	s	%_vol._	ppm													
700	HDPE	197	0	0.00	0.00	0.00	0.00	0.00	0.00	0.00	0.00	0.00	0.00	0.00	0.00	0.00	0.00	0.00
			7	0.15	91.71	1.31	0.00	0.00	2.01	0.00	1.75	0.00	0.00	0.00	0.00	0.00	0.00	0.00
			14	3.34	982.78	5.70	0.00	0.00	4.29	0.00	3.00	0.00	0.00	0.00	0.00	0.00	0.00	0.00
			21	3.10	1720.08	10.80	0.00	0.00	4.95	0.00	3.68	0.00	0.00	0.00	0.00	0.00	0.00	0.00
			28	1.31	1584.29	17.23	0.00	0.00	11.30	0.00	5.44	0.00	0.07	0.00	0.00	0.00	0.00	0.00
			35	0.56	453.05	5.79	0.00	0.00	0.81	0.00	1.35	0.00	0.00	0.00	0.00	0.00	0.00	0.00
			42	0.18	113.44	1.75	0.00	0.00	0.00	0.00	1.19	0.00	0.00	0.00	0.00	0.00	0.00	0.00
			49	0.06	30.03	2.23	0.00	0.00	0.00	0.00	0.29	0.00	0.00	0.00	0.00	0.00	0.00	0.00
			56	0.03	8.33	1.16	0.00	0.00	0.00	0.00	0.00	0.00	0.00	0.00	0.00	0.00	0.00	0.00
			63	0.03	2.45	1.01	0.00	0.00	0.00	0.00	0.00	0.00	0.00	0.00	0.00	0.00	0.00	0.00

		303	0	0.00	0.00	0.00	0.00	0.00	0.00	0.00	0.00	0.00	0.00	0.00	0.00	0.00	0.00	0.00
			7	0.07	84.74	2.20	0.00	0.00	0.00	0.00	3.03	0.00	0.00	0.26	1.26	0.00	0.00	0.00
			14	3.69	1181.93	34.78	0.00	0.00	25.04	0.00	8.31	0.00	0.00	5.00	3.53	0.00	0.00	0.00
			21	5.85	2559.33	58.50	0.00	0.00	33.85	0.00	9.61	0.00	0.00	7.33	4.21	0.00	0.00	0.00
			28	2.20	2202.75	24.52	0.00	0.00	10.40	0.00	4.51	0.00	0.00	2.69	3.46	0.00	0.00	0.00
			35	0.92	833.83	9.27	0.00	0.00	0.00	0.00	3.44	0.00	0.00	1.10	1.46	0.00	0.00	0.00
			42	0.26	215.17	2.79	0.00	0.00	0.00	0.00	1.36	0.00	0.00	0.77	1.41	0.00	0.00	0.00
			49	0.05	57.63	1.15	0.00	0.00	0.00	0.00	0.30	0.00	0.00	0.00	0.00	0.00	0.00	0.00
			56	0.02	14.40	1.16	0.00	0.00	0.00	0.00	0.00	0.00	0.00	0.00	0.00	0.00	0.00	0.00
			63	0.00	3.87	0.13	0.00	0.00	0.00	0.00	0.00	0.00	0.00	0.00	0.00	0.00	0.00	0.00

		100	0	0.00	0.00	0.00	0.00	0.00	0.00	0.00	0.00	0.00	0.00	0.00	0.00	0.00	0.00	0.00
			7	0.12	257.37	5.20	0.00	0.00	0.00	0.00	0.00	0.00	0.00	0.00	0.00	0.00	0.00	0.00
			14	1.18	959.37	8.69	0.00	0.00	0.00	0.00	0.00	0.00	0.00	0.00	0.00	0.00	0.00	0.00
			21	1.86	1033.41	8.85	0.00	0.00	0.00	0.00	0.00	0.00	0.00	0.00	0.00	0.00	0.00	0.00
			28	0.75	624.82	8.99	0.00	0.00	0.00	0.00	0.00	0.00	0.00	0.00	0.00	0.00	0.00	0.00
			35	0.24	167.06	2.54	0.00	0.00	0.00	0.00	0.00	0.00	0.00	0.00	0.00	0.00	0.00	0.00
			42	0.07	45.27	1.25	0.00	0.00	0.00	0.00	0.00	0.00	0.00	0.00	0.00	0.00	0.00	0.00
			49	0.03	9.93	1.10	0.00	0.00	0.00	0.00	0.00	0.00	0.00	0.00	0.00	0.00	0.00	0.00
56	0.00		2.69	0.82	0.00	0.00	0.00	0.00	0.00	0.00	0.00	0.00	0.00	0.00	0.00	0.00
63	0.00		0.33	0.00	0.00	0.00	0.00	0.00	0.00	0.00	0.00	0.00	0.00	0.00	0.00	0.00

In the related research article [1] an innovative fluidised bed made from cenospheres has been used. The use of cenospheres (0.845 g.cm^3^) made it possible to obtain a bed with a density comparable or smaller to that of polyethylene (0.92-0.96 g/cm^3^). Such innovation had a crucial influence on combustion because diffusion combustion in the freeboard was eliminated. Even higher fuel conversion at lower temperatures and the elimination of explosions have been achieved by the catalytic combustion process using Fe_2_O_3_-cenospheres. The composition of flue gas during PE combustion in fluidised beds of cenospheres and Fe_2_O_3_-cenospheres was monitored online by FTIR analyser.

**Table 11:** Composition of flue gases during PE combustion in a fluidised bed of cenospheres at 800 °C.

Temp.	Polymer	Mass	Time	CO2	CO	CH4	C2H6	C3H8	C2H4	C4+	HCHO	CH3CHO	Acrolein	C2H2	C6H6	CH3OH	CH3COOH	CH3COCH3
°C	-	mg	s	%_vol._	ppm													
800	PE	203	0	0.00	0.00	0.00	0.00	0.00	0.00	0.00	0.00	0.00	0.00	0.00	0.00	0.00	0.00	0.00
			7	0.29	6.32	0.00	0.00	0.00	0.00	0.00	0.00	0.00	0.00	0.00	0.00	0.00	0.00	0.00
			14	3.96	84.36	0.00	0.00	0.00	0.00	0.00	0.00	0.00	0.00	0.00	0.00	0.00	0.00	0.00
			21	3.34	51.36	0.00	0.00	0.00	0.00	0.00	0.00	0.00	0.00	0.00	0.00	0.00	0.00	0.00
			28	1.06	13.67	0.00	0.00	0.00	0.00	0.00	0.00	0.00	0.00	0.00	0.00	0.00	0.00	0.00
			35	0.33	0.99	0.00	0.00	0.00	0.00	0.00	0.00	0.00	0.00	0.00	0.00	0.00	0.00	0.00
			42	0.10	0.00	0.00	0.00	0.00	0.00	0.00	0.00	0.00	0.00	0.00	0.00	0.00	0.00	0.00
			49	0.03	0.03	0.00	0.00	0.00	0.00	0.00	0.00	0.00	0.00	0.00	0.00	0.00	0.00	0.00
			56	0.03	0.00	0.00	0.00	0.00	0.00	0.00	0.00	0.00	0.00	0.00	0.00	0.00	0.00	0.00
			63	0.00	0.00	0.00	0.00	0.00	0.00	0.00	0.00	0.00	0.00	0.00	0.00	0.00	0.00	0.00

		200	0	0.00	0.00	0.00	0.00	0.00	0.00	0.00	0.00	0.00	0.00	0.00	0.00	0.00	0.00	0.00
			7	0.04	3.82	0.00	0.00	0.00	0.00	0.00	0.00	0.00	0.00	0.00	0.00	0.00	0.00	0.00
			14	2.73	34.65	0.00	0.00	0.00	0.00	0.00	0.00	0.00	0.00	0.00	0.00	0.00	0.00	0.00
			21	4.06	34.14	0.00	0.00	0.00	0.00	0.00	0.00	0.00	0.00	0.00	0.00	0.00	0.00	0.00
			28	1.51	12.58	0.00	0.00	0.00	0.00	0.00	0.00	0.00	0.00	0.00	0.00	0.00	0.00	0.00
			35	0.48	4.16	0.00	0.00	0.00	0.00	0.00	0.00	0.00	0.00	0.00	0.00	0.00	0.00	0.00
			42	0.13	3.75	0.00	0.00	0.00	0.00	0.00	0.00	0.00	0.00	0.00	0.00	0.00	0.00	0.00
			49	0.04	2.24	0.00	0.00	0.00	0.00	0.00	0.00	0.00	0.00	0.00	0.00	0.00	0.00	0.00
			56	0.01	1.80	0.00	0.00	0.00	0.00	0.00	0.00	0.00	0.00	0.00	0.00	0.00	0.00	0.00
			63	0.00	2.37	0.00	0.00	0.00	0.00	0.00	0.00	0.00	0.00	0.00	0.00	0.00	0.00	0.00

		98	0	0.00	0.00	0.00	0.00	0.00	0.00	0.00	0.00	0.00	0.00	0.00	0.00	0.00	0.00	0.00
			7	0.25	1.75	0.00	0.00	0.00	0.00	0.00	0.00	0.00	0.00	0.00	0.00	0.00	0.00	0.00
			14	2.25	9.01	0.00	0.00	0.00	0.00	0.00	0.00	0.00	0.00	0.00	0.00	0.00	0.00	0.00
			21	1.24	4.18	0.00	0.00	0.00	0.00	0.00	0.00	0.00	0.00	0.00	0.00	0.00	0.00	0.00
			28	0.42	0.63	0.00	0.00	0.00	0.00	0.00	0.00	0.00	0.00	0.00	0.00	0.00	0.00	0.00
			35	0.12	0.00	0.00	0.00	0.00	0.00	0.00	0.00	0.00	0.00	0.00	0.00	0.00	0.00	0.00
			42	0.04	0.00	0.00	0.00	0.00	0.00	0.00	0.00	0.00	0.00	0.00	0.00	0.00	0.00	0.00
			49	0.01	0.00	0.00	0.00	0.00	0.00	0.00	0.00	0.00	0.00	0.00	0.00	0.00	0.00	0.00
56	0.01		0.00	0.00	0.00	0.00	0.00	0.00	0.00	0.00	0.00	0.00	0.00	0.00	0.00	0.00
63	0.01		0.00	0.00	0.00	0.00	0.00	0.00	0.00	0.00	0.00	0.00	0.00	0.00	0.00	0.00

**Table 12:** Composition of flue gases during LLDPE combustion in a fluidised bed of cenospheres at 800 °C.

Temp.	Polymer	Mass	Time	CO2	CO	CH4	C2H6	C3H8	C2H4	C4+	HCHO	CH3CHO	Acrolein	C2H2	C6H6	CH3OH	CH3COOH	CH3COCH3
°C	-	mg	s	%_vol._	ppm													
800	LLDPE	204	0	0.00	0.00	0.00	0.00	0.00	0.00	0.00	0.00	0.00	0.00	0.00	0.00	0.00	0.00	0.00
			7	1.08	16.82	0.00	0.00	0.00	0.00	0.00	0.00	0.00	0.00	0.00	0.00	0.00	0.00	0.00
			14	4.82	63.74	0.00	0.00	0.00	0.00	0.00	0.00	0.00	0.00	0.00	0.00	0.00	0.00	0.00
			21	2.23	22.44	0.00	0.00	0.00	0.00	0.00	0.00	0.00	0.00	0.00	0.00	0.00	0.00	0.00
			28	0.75	5.94	0.00	0.00	0.00	0.00	0.00	0.00	0.00	0.00	0.00	0.00	0.00	0.00	0.00
			35	0.25	2.30	0.00	0.00	0.00	0.00	0.00	0.00	0.00	0.00	0.00	0.00	0.00	0.00	0.00
			42	0.07	0.00	0.00	0.00	0.00	0.00	0.00	0.00	0.00	0.00	0.00	0.00	0.00	0.00	0.00
			49	0.01	0.00	0.00	0.00	0.00	0.00	0.00	0.00	0.00	0.00	0.00	0.00	0.00	0.00	0.00
			56	0.01	0.00	0.00	0.00	0.00	0.00	0.00	0.00	0.00	0.00	0.00	0.00	0.00	0.00	0.00
			63	0.00	0.40	0.00	0.00	0.00	0.00	0.00	0.00	0.00	0.00	0.00	0.00	0.00	0.00	0.00

		205	0	0.00	0.00	0.00	0.00	0.00	0.00	0.00	0.00	0.00	0.00	0.00	0.00	0.00	0.00	0.00
			7	0.08	4.13	0.00	0.00	0.00	0.00	0.00	0.00	0.00	0.00	0.00	0.00	0.00	0.00	0.00
			14	3.52	68.51	0.00	0.00	0.00	0.00	0.00	0.00	0.00	0.00	0.00	0.00	0.00	0.00	0.00
			21	3.99	66.65	0.00	0.00	0.00	0.00	0.00	0.00	0.00	0.00	0.00	0.00	0.00	0.00	0.00
			28	1.37	18.16	0.00	0.00	0.00	0.00	0.00	0.00	0.00	0.00	0.00	0.00	0.00	0.00	0.00
			35	0.44	4.82	0.00	0.00	0.00	0.00	0.00	0.00	0.00	0.00	0.00	0.00	0.00	0.00	0.00
			42	0.12	0.19	0.00	0.00	0.00	0.00	0.00	0.00	0.00	0.00	0.00	0.00	0.00	0.00	0.00
			49	0.04	0.46	0.00	0.00	0.00	0.00	0.00	0.00	0.00	0.00	0.00	0.00	0.00	0.00	0.00
			56	0.00	0.00	0.00	0.00	0.00	0.00	0.00	0.00	0.00	0.00	0.00	0.00	0.00	0.00	0.00
			63	0.03	0.17	0.00	0.00	0.00	0.00	0.00	0.00	0.00	0.00	0.00	0.00	0.00	0.00	0.00

		101	0	0.00	0.00	0.00	0.00	0.00	0.00	0.00	0.00	0.00	0.00	0.00	0.00	0.00	0.00	0.00
			7	0.76	11.81	0.00	0.00	0.00	0.00	0.00	0.00	0.00	0.00	0.00	0.00	0.00	0.00	0.00
			14	2.21	19.18	0.00	0.00	0.00	0.00	0.00	0.00	0.00	0.00	0.00	0.00	0.00	0.00	0.00
			21	1.05	7.45	0.00	0.00	0.00	0.00	0.00	0.00	0.00	0.00	0.00	0.00	0.00	0.00	0.00
			28	0.31	1.14	0.00	0.00	0.00	0.00	0.00	0.00	0.00	0.00	0.00	0.00	0.00	0.00	0.00
			35	0.09	0.68	0.00	0.00	0.00	0.00	0.00	0.00	0.00	0.00	0.00	0.00	0.00	0.00	0.00
			42	0.01	0.00	0.00	0.00	0.00	0.00	0.00	0.00	0.00	0.00	0.00	0.00	0.00	0.00	0.00
			49	0.01	0.00	0.00	0.00	0.00	0.00	0.00	0.00	0.00	0.00	0.00	0.00	0.00	0.00	0.00
56	0.00		0.00	0.00	0.00	0.00	0.00	0.00	0.00	0.00	0.00	0.00	0.00	0.00	0.00	0.00
63	0.00		0.00	0.00	0.00	0.00	0.00	0.00	0.00	0.00	0.00	0.00	0.00	0.00	0.00	0.00

**Table 13:** Composition of flue gases during HDPE combustion in a fluidised bed of cenospheres at 800 °C.

Temp.	Polymer	Mass	Time	CO2	CO	CH4	C2H6	C3H8	C2H4	C4+	HCHO	CH3CHO	Acrolein	C2H2	C6H6	CH3OH	CH3COOH	CH3COCH3
°C	-	mg	s	%_vol._	ppm													
800	HDPE	204	0	0.00	0.00	0.00	0.00	0.00	0.00	0.00	0.00	0.00	0.00	0.00	0.00	0.00	0.00	0.00
			7	0.12	2.25	0.00	0.00	0.00	0.00	0.00	0.00	0.00	0.00	0.00	0.00	0.00	0.00	0.00
			14	1.93	21.44	0.00	0.00	0.00	0.00	0.00	0.00	0.00	0.00	0.00	0.00	0.00	0.00	0.00
			21	4.06	46.76	0.00	0.00	0.00	0.00	0.00	0.00	0.00	0.00	0.00	0.00	0.00	0.00	0.00
			28	2.16	23.18	0.00	0.00	0.00	0.00	0.00	0.00	0.00	0.00	0.00	0.00	0.00	0.00	0.00
			35	0.69	5.09	0.00	0.00	0.00	0.00	0.00	0.00	0.00	0.00	0.00	0.00	0.00	0.00	0.00
			42	0.23	0.32	0.00	0.00	0.00	0.00	0.00	0.00	0.00	0.00	0.00	0.00	0.00	0.00	0.00
			49	0.05	0.00	0.00	0.00	0.00	0.00	0.00	0.00	0.00	0.00	0.00	0.00	0.00	0.00	0.00
			56	0.01	0.00	0.00	0.00	0.00	0.00	0.00	0.00	0.00	0.00	0.00	0.00	0.00	0.00	0.00

		300	0	0.00	0.00	0.00	0.00	0.00	0.00	0.00	0.00	0.00	0.00	0.00	0.00	0.00	0.00	0.00
			7	0.60	11.22	0.91	0.00	0.00	2.59	0.00	0.00	0.00	0.00	0.00	0.00	0.00	0.00	0.00
			14	5.36	460.18	25.20	0.00	0.00	21.58	0.00	0.00	0.00	0.00	0.00	0.00	0.00	0.00	0.00
			21	5.49	465.60	24.91	0.00	0.00	18.93	0.00	0.00	0.00	0.00	0.00	0.00	0.00	0.00	0.00
			28	1.89	113.65	6.20	0.00	0.00	0.00	0.00	0.00	0.00	0.00	0.00	0.00	0.00	0.00	0.00
			35	0.64	31.22	1.01	0.00	0.00	0.00	0.00	0.00	0.00	0.00	0.00	0.00	0.00	0.00	0.00
			42	0.20	7.05	0.78	0.00	0.00	0.00	0.00	0.00	0.00	0.00	0.00	0.00	0.00	0.00	0.00
			49	0.06	3.52	0.37	0.00	0.00	0.00	0.00	0.00	0.00	0.00	0.00	0.00	0.00	0.00	0.00
			56	0.03	3.15	0.00	0.00	0.00	0.00	0.00	0.00	0.00	0.00	0.05	0.00	0.00	0.00	0.00
			63	0.00	1.53	0.00	0.00	0.00	0.00	0.00	0.00	0.00	0.00	0.00	0.00	0.00	0.00	0.00

		103	0	0.00	0.00	0.00	0.00	0.00	0.00	0.00	0.00	0.00	0.00	0.00	0.00	0.00	0.00	0.00
			7	0.65	3.07	0.00	0.00	0.00	0.00	0.00	0.00	0.00	0.00	0.00	0.00	0.00	0.00	0.00
			14	2.31	15.52	0.00	0.00	0.00	0.00	0.00	0.00	0.00	0.00	0.00	0.00	0.00	0.00	0.00
			21	1.10	7.56	0.00	0.00	0.00	0.00	0.00	0.00	0.00	0.00	0.00	0.00	0.00	0.00	0.00
			28	0.37	1.61	0.00	0.00	0.00	0.00	0.00	0.00	0.00	0.00	0.00	0.00	0.00	0.00	0.00
			35	0.13	0.00	0.00	0.00	0.00	0.00	0.00	0.00	0.00	0.00	0.00	0.00	0.00	0.00	0.00
42	0.03		0.00	0.00	0.00	0.00	0.00	0.00	0.00	0.00	0.00	0.00	0.00	0.00	0.00	0.00
49	0.00		0.00	0.00	0.00	0.00	0.00	0.00	0.00	0.00	0.00	0.00	0.00	0.00	0.00	0.00

**Table 14:** Composition of flue gases during PE combustion in a fluidised bed of cenospheres at 900 °C.

Temp.	Polymer	Mass	Time	CO2	CO	CH4	C2H6	C3H8	C2H4	C4+	HCHO	CH3CHO	Acrolein	C2H2	C6H6	CH3OH	CH3COOH	CH3COCH3
°C	-	mg	s	%_vol._	ppm													
900	PE	200	0	0.00	0.00	0.00	0.00	0.00	0.00	0.00	0.00	0.00	0.00	0.00	0.00	0.00	0.00	0.00
			7	2.96	177.57	12.45	0.00	0.00	4.48	0.00	0.00	0.00	0.00	6.41	0.00	0.00	0.00	0.00
			14	4.03	458.76	21.70	0.00	0.00	10.62	0.00	0.00	0.00	0.00	9.59	0.00	0.00	0.00	0.00
			21	1.23	104.27	5.22	0.00	0.00	2.16	0.00	0.00	0.00	0.00	4.36	0.00	0.00	0.00	0.00
			28	0.41	22.26	0.11	0.00	0.00	0.15	0.00	0.00	0.00	0.00	2.27	0.00	0.00	0.00	0.00
			35	0.10	3.36	0.00	0.00	0.00	0.23	0.00	0.00	0.00	0.00	1.48	0.00	0.00	0.00	0.00
			42	0.04	0.00	0.00	0.00	0.00	0.00	0.00	0.00	0.00	0.00	0.00	0.00	0.00	0.00	0.00
			49	0.00	0.00	0.00	0.00	0.00	0.00	0.00	0.00	0.00	0.00	0.00	0.00	0.00	0.00	0.00

		204	0	0.00	0.00	0.00	0.00	0.00	0.00	0.00	0.00	0.00	0.00	0.00	0.00	0.00	0.00	0.00
			7	0.03	4.73	0.00	0.00	0.00	0.36	0.00	0.18	0.00	0.00	0.20	0.00	0.00	0.00	0.00
			14	1.25	46.91	3.08	0.00	0.00	0.77	0.00	1.60	0.00	0.00	1.21	0.00	0.00	0.00	0.00
			21	5.39	567.64	36.48	0.00	0.00	26.20	0.00	8.12	0.00	0.00	13.79	0.00	0.00	0.00	0.00
			28	1.86	143.27	9.83	0.00	0.00	3.25	0.00	1.73	0.00	0.00	3.80	0.00	0.00	0.00	0.00
			35	0.65	35.36	2.39	0.00	0.00	0.00	0.00	1.32	0.00	0.00	1.77	0.00	0.00	0.00	0.00
			42	0.22	9.39	0.01	0.00	0.00	0.00	0.00	0.00	0.00	0.00	0.18	0.00	0.00	0.00	0.00
			49	0.07	3.71	0.17	0.00	0.00	0.00	0.00	0.12	0.00	0.00	0.00	0.00	0.00	0.00	0.00
			56	0.04	1.56	0.00	0.00	0.00	0.00	0.00	0.00	0.00	0.00	0.00	0.00	0.00	0.00	0.00
			63	0.03	0.00	0.00	0.00	0.00	0.00	0.00	0.00	0.00	0.00	0.00	0.00	0.00	0.00	0.00

		102	0	0.00	0.00	0.00	0.00	0.00	0.00	0.00	0.00	0.00	0.00	0.00	0.00	0.00	0.00	0.00
			7	1.19	0.00	0.00	0.00	0.00	0.00	0.00	0.00	0.00	0.00	0.00	0.00	0.00	0.00	0.00
			14	2.27	0.77	0.00	0.00	0.00	0.00	0.00	0.00	0.00	0.00	0.00	0.00	0.00	0.00	0.00
			21	0.78	2.29	0.00	0.00	0.00	0.00	0.00	0.00	0.00	0.00	0.00	0.00	0.00	0.00	0.00
			28	0.26	1.31	0.00	0.00	0.00	0.00	0.00	0.00	0.00	0.00	0.00	0.00	0.00	0.00	0.00
			35	0.10	1.53	0.00	0.00	0.00	0.00	0.00	0.00	0.00	0.00	0.00	0.00	0.00	0.00	0.00
			42	0.05	0.33	0.00	0.00	0.00	0.00	0.00	0.00	0.00	0.00	0.00	0.00	0.00	0.00	0.00
49	0.02		0.15	0.00	0.00	0.00	0.00	0.00	0.00	0.00	0.00	0.00	0.00	0.00	0.00	0.00
56	0.00		0.00	0.00	0.00	0.00	0.00	0.00	0.00	0.00	0.00	0.00	0.00	0.00	0.00	0.00

**Table 15:** Composition of flue gases during LLDPE combustion in a fluidised bed of cenospheres at 900 °C.

Temp.	Polymer	Mass	Time	CO2	CO	CH4	C2H6	C3H8	C2H4	C4+	HCHO	CH3CHO	Acrolein	C2H2	C6H6	CH3OH	CH3COOH	CH3COCH3
°C	-	mg	s	%_vol._	ppm													
900	LLDPE	202	0	0.00	0.00	0.00	0.00	0.00	0.00	0.00	0.00	0.00	0.00	0.00	0.00	0.00	0.00	0.00
			7	1.42	32.51	3.64	0.00	0.00	0.00	0.00	0.00	0.00	0.00	0.00	0.00	0.00	0.00	0.00
			14	5.27	240.79	18.30	0.00	0.00	0.00	0.00	0.00	0.00	0.00	0.00	0.00	0.00	0.00	0.00
			21	1.94	61.87	5.25	0.00	0.00	0.00	0.00	0.00	0.00	0.00	0.00	0.00	0.00	0.00	0.00
			28	0.62	15.70	0.52	0.00	0.00	0.00	0.00	0.00	0.00	0.00	0.00	0.00	0.00	0.00	0.00
			35	0.20	4.98	0.00	0.00	0.00	0.00	0.00	0.00	0.00	0.00	0.00	0.00	0.00	0.00	0.00
			42	0.08	1.73	0.00	0.00	0.00	0.00	0.00	0.00	0.00	0.00	0.00	0.00	0.00	0.00	0.00
			49	0.03	2.08	0.00	0.00	0.00	0.00	0.00	0.00	0.00	0.00	0.00	0.00	0.00	0.00	0.00
			56	0.01	2.09	0.00	0.00	0.00	0.00	0.00	0.00	0.00	0.00	0.00	0.00	0.00	0.00	0.00

		200	0	0.00	0.00	0.00	0.00	0.00	0.00	0.00	0.00	0.00	0.00	0.00	0.00	0.00	0.00	0.00
			7	0.16	0.00	0.00	0.00	0.00	0.00	0.00	0.00	0.00	0.00	0.00	0.00	0.00	0.00	0.00
			14	4.49	0.00	0.00	0.00	0.00	0.00	0.00	0.00	0.00	0.00	0.00	0.00	0.00	0.00	0.00
			21	3.13	0.00	0.00	0.00	0.00	0.00	0.00	0.00	0.00	0.00	0.00	0.00	0.00	0.00	0.00
			28	1.07	0.00	0.00	0.00	0.00	0.00	0.00	0.00	0.00	0.00	0.00	0.00	0.00	0.00	0.00
			35	0.34	0.00	0.00	0.00	0.00	0.00	0.00	0.00	0.00	0.00	0.00	0.00	0.00	0.00	0.00
			42	0.13	0.00	0.00	0.00	0.00	0.00	0.00	0.00	0.00	0.00	0.00	0.00	0.00	0.00	0.00
			49	0.04	0.00	0.00	0.00	0.00	0.00	0.00	0.00	0.00	0.00	0.00	0.00	0.00	0.00	0.00
			56	0.00	0.00	0.00	0.00	0.00	0.00	0.00	0.00	0.00	0.00	0.00	0.00	0.00	0.00	0.00

		100	0	0.00	0.00	0.00	0.00	0.00	0.00	0.00	0.00	0.00	0.00	0.00	0.00	0.00	0.00	0.00
			7	0.04	0.00	0.00	0.00	0.00	0.00	0.00	0.00	0.00	0.00	0.00	0.00	0.00	0.00	0.00
			14	1.88	0.00	0.00	0.00	0.00	0.00	0.00	0.00	0.00	0.00	0.00	0.00	0.00	0.00	0.00
			21	1.81	0.00	0.00	0.00	0.00	0.00	0.00	0.00	0.00	0.00	0.00	0.00	0.00	0.00	0.00
			28	0.62	0.00	0.00	0.00	0.00	0.00	0.00	0.00	0.00	0.00	0.00	0.00	0.00	0.00	0.00
			35	0.21	0.00	0.00	0.00	0.00	0.00	0.00	0.00	0.00	0.00	0.00	0.00	0.00	0.00	0.00
			42	0.07	0.00	0.00	0.00	0.00	0.00	0.00	0.00	0.00	0.00	0.00	0.00	0.00	0.00	0.00
49	0.03		0.00	0.00	0.00	0.00	0.00	0.00	0.00	0.00	0.00	0.00	0.00	0.00	0.00	0.00
56	0.03		0.00	0.00	0.00	0.00	0.00	0.00	0.00	0.00	0.00	0.00	0.00	0.00	0.00	0.00

This work presents data from a real fluidised bed combustor. The data presents the composition of flue gas during polyethylene combustion. The dataset contains 31 Tables. Table 1 contains the parameters of the reactor, bed material, and fluidising gas. Table 2, Table 3, Table 4, Table 5, Table 6, Table 7, Table 8, Table 9, Table 10, Table 11, Table 12, Table 13, Table 14, Table 15, Table 16, Table 17, Table 18, Table 19, Table 20, Table 21, Table 22, Table 23, Table 24, Table 25, Table 26, Table 27, Table 28, Table 29, Table 30, Table 31 provide information on the change in concentration of emitted components as a function of time during, PE, LLDPE, HDPE fluidised bed combustion.

**Table 16:** Composition of flue gases during HDPE combustion in a fluidised bed of cenospheres at 900 °C.

Temp.	Polymer	Mass	Time	CO2	CO	CH4	C2H6	C3H8	C2H4	C4+	HCHO	CH3CHO	Acrolein	C2H2	C6H6	CH3OH	CH3COOH	CH3COCH3
°C	-	mg	s	%_vol._	ppm													
900	HDPE	198	0	0.00	0.00	0.00	0.00	0.00	0.00	0.00	0.00	0.00	0.00	0.00	0.00	0.00	0.00	0.00
			7	0.35	0.00	0.31	0.00	0.00	0.00	0.00	0.00	0.00	0.00	0.00	0.00	0.00	0.00	0.00
			14	4.91	704.09	53.72	0.00	0.00	0.00	0.00	0.00	0.00	0.00	0.00	0.00	0.00	0.00	0.00
			21	2.50	207.94	15.54	0.00	0.00	0.00	0.00	0.00	0.00	0.00	0.00	0.00	0.00	0.00	0.00
			28	0.84	50.44	4.48	0.00	0.00	0.00	0.00	0.00	0.00	0.00	0.00	0.00	0.00	0.00	0.00
			35	0.26	11.76	1.07	0.00	0.00	0.00	0.00	0.00	0.00	0.00	0.00	0.00	0.00	0.00	0.00
			42	0.09	3.53	0.23	0.00	0.00	0.00	0.00	0.00	0.00	0.00	0.00	0.00	0.00	0.00	0.00
			49	0.06	1.56	0.00	0.00	0.00	0.00	0.00	0.00	0.00	0.00	0.00	0.00	0.00	0.00	0.00
			56	0.01	0.34	0.00	0.00	0.00	0.00	0.00	0.00	0.00	0.00	0.00	0.00	0.00	0.00	0.00
			63	0.00	0.00	0.00	0.00	0.00	0.00	0.00	0.00	0.00	0.00	0.00	0.00	0.00	0.00	0.00

		203	0	0.00	0.00	0.00	0.00	0.00	0.00	0.00	0.00	0.00	0.00	0.00	0.00	0.00	0.00	0.00
			7	0.03	0.00	0.00	0.00	0.00	0.00	0.00	0.00	0.00	0.00	0.00	0.00	0.00	0.00	0.00
			14	3.44	1.28	0.00	0.00	0.00	0.00	0.00	0.00	0.00	0.00	0.00	0.00	0.00	0.00	0.00
			21	3.67	13.58	0.00	0.00	0.00	0.00	0.00	0.00	0.00	0.00	0.00	0.00	0.00	0.00	0.00
			28	1.29	4.81	0.00	0.00	0.00	0.00	0.00	0.00	0.00	0.00	0.00	0.00	0.00	0.00	0.00
			35	0.41	1.98	0.00	0.00	0.00	0.00	0.00	0.00	0.00	0.00	0.00	0.00	0.00	0.00	0.00
			42	0.11	0.00	0.00	0.00	0.00	0.00	0.00	0.00	0.00	0.00	0.00	0.00	0.00	0.00	0.00
			49	0.00	0.00	0.00	0.00	0.00	0.00	0.00	0.00	0.00	0.00	0.00	0.00	0.00	0.00	0.00

		101	0	0.00	0.00	0.00	0.00	0.00	0.00	0.00	0.00	0.00	0.00	0.00	0.00	0.00	0.00	0.00
			7	1.07	0.23	0.00	0.00	0.00	0.00	0.00	0.00	0.00	0.00	0.00	0.00	0.00	0.00	0.00
			14	2.01	1.93	0.00	0.00	0.00	0.00	0.00	0.00	0.00	0.00	0.00	0.00	0.00	0.00	0.00
			21	1.05	1.62	0.00	0.00	0.00	0.00	0.00	0.00	0.00	0.00	0.00	0.00	0.00	0.00	0.00
			28	0.33	1.04	0.00	0.00	0.00	0.00	0.00	0.00	0.00	0.00	0.00	0.00	0.00	0.00	0.00
			35	0.12	0.45	0.00	0.00	0.00	0.00	0.00	0.00	0.00	0.00	0.00	0.00	0.00	0.00	0.00
			42	0.02	0.37	0.00	0.00	0.00	0.00	0.00	0.00	0.00	0.00	0.00	0.00	0.00	0.00	0.00
49	0.00		0.23	0.00	0.00	0.00	0.00	0.00	0.00	0.00	0.00	0.00	0.00	0.00	0.00	0.00
56	0.00		0.00	0.00	0.00	0.00	0.00	0.00	0.00	0.00	0.00	0.00	0.00	0.00	0.00	0.00

**Table 17:** Composition of flue gases during PE combustion in a fluidised bed of Fe_2_O_3_-cenospheres at 500 °C.

Temp.	Polymer	Mass	Time	CO2	CO	CH4	C2H6	C3H8	C2H4	C4+	HCHO	CH3CHO	Acrolein	C2H2	C6H6	CH3OH	CH3COOH	CH3COCH3
°C	-	mg	s	%_vol._	ppm													
500	PE	203	0	0.00	0.00	0.00	0.00	0.00	0.00	0.00	0.00	0.00	0.00	0.00	0.00	0.00	0.00	0.00
			7	0.01	1.48	0.39	0.00	0.00	0.00	0.00	0.00	0.13	0.00	0.00	0.00	0.00	0.00	0.00
			14	0.21	111.88	2.06	0.00	0.00	7.35	4.23	0.88	0.62	0.00	0.00	1.40	0.00	0.00	0.00
			21	0.79	456.93	14.10	0.00	0.00	35.16	36.72	9.38	3.71	0.00	0.03	9.22	0.00	0.00	0.00
			29	1.42	734.92	20.30	0.00	0.00	72.11	93.55	27.42	28.65	0.00	0.00	21.77	0.00	0.00	0.00
			36	1.55	865.00	28.43	0.00	0.00	85.36	134.57	44.75	79.31	0.00	9.85	32.46	0.00	0.00	0.00
			43	0.94	373.03	30.04	0.00	0.00	29.37	68.10	17.25	19.56	0.00	4.09	14.11	0.00	0.00	0.00
			50	0.42	125.37	15.18	0.00	0.00	7.63	30.31	2.90	4.83	0.00	2.24	9.48	0.00	0.00	0.00
			57	0.25	55.48	7.46	0.00	0.00	2.63	16.02	0.50	1.40	0.00	0.00	5.47	0.00	0.00	0.00
			64	0.14	27.55	4.34	0.00	0.00	2.75	10.14	0.00	0.00	0.00	0.00	3.50	0.00	0.00	0.00
			71	0.09	16.85	4.22	0.00	0.00	1.14	7.29	0.00	0.00	0.00	0.00	2.43	0.00	0.00	0.00
			79	0.06	9.50	2.55	0.00	0.00	0.38	5.38	0.00	0.00	0.00	0.00	0.90	0.00	0.00	0.00

		301	0	0.00	0.00	0.00	0.00	0.00	0.00	0.00	0.00	0.00	0.00	0.00	0.00	0.00	0.00	0.00
			7	0.18	85.69	1.08	0.00	0.00	2.27	2.59	1.12	0.00	0.00	0.00	0.58	0.00	0.00	0.00
			15	0.98	609.67	10.60	0.00	0.00	48.39	48.10	14.90	0.00	0.00	5.65	11.07	0.00	0.00	0.00
			22	2.02	1537.52	40.04	0.00	0.00	142.21	206.41	67.54	90.78	0.00	22.17	45.94	0.00	0.00	0.00
			29	1.97	1509.04	50.49	0.00	0.00	152.55	271.60	86.93	171.19	0.00	18.70	70.64	0.00	0.00	0.00
			36	1.44	644.70	21.97	0.00	0.00	66.12	134.84	33.39	88.07	0.00	7.97	26.76	0.00	0.00	0.00
			43	0.74	265.94	23.81	0.00	0.00	19.93	61.49	7.88	37.83	0.00	1.56	15.81	0.00	0.00	0.00
			50	0.49	144.95	19.09	0.00	0.00	9.86	33.06	1.83	0.00	0.00	0.00	6.57	0.00	0.00	0.00
			58	0.32	73.12	8.53	0.00	0.00	0.00	19.08	0.61	0.00	0.00	0.00	5.31	0.00	0.00	0.00
			65	0.15	31.86	5.94	0.00	0.00	0.00	12.73	0.00	0.00	0.00	0.00	5.51	0.00	0.00	0.00
			72	0.08	13.90	4.30	0.00	0.00	0.00	8.72	0.00	0.00	0.00	0.00	2.46	0.00	0.00	0.00
			79	0.04	5.51	3.04	0.00	0.00	0.00	7.17	0.00	0.00	0.00	0.00	1.62	0.00	0.00	0.00

		101	0	0.00	0.00	0.00	0.00	0.00	0.00	0.00	0.00	0.00	0.00	0.00	0.00	0.00	0.00	0.00
			7	0.02	8.52	0.02	0.00	0.00	0.00	0.00	0.51	0.00	0.00	0.00	0.30	0.00	0.00	0.00
			14	0.29	170.52	3.89	0.00	0.00	12.58	6.52	1.97	0.00	0.00	0.00	3.72	0.00	0.00	0.00
			22	0.67	425.59	15.63	0.00	0.00	38.95	36.63	11.89	0.00	0.00	4.42	10.30	3.95	0.00	0.00
			29	1.10	688.72	14.73	0.00	0.00	62.94	88.63	29.63	43.41	0.00	9.27	19.82	8.12	0.00	0.00
			36	0.76	421.25	8.10	0.00	0.00	40.80	64.32	18.98	7.59	0.00	5.07	21.36	0.01	0.00	0.00
			43	0.31	125.53	8.40	0.00	0.00	6.90	25.89	6.29	0.00	0.00	0.16	7.37	0.00	0.00	0.00
			50	0.09	31.62	2.58	0.00	0.00	2.86	8.57	0.79	0.00	0.00	0.00	3.00	0.00	0.00	0.00
57	0.00		5.93	0.76	0.00	0.00	0.00	2.28	0.79	0.00	0.00	0.00	1.78	0.00	0.00	0.00
64	0.00		0.26	0.43	0.00	0.00	0.00	0.00	0.35	0.00	0.00	0.00	0.84	0.00	0.00	0.00

**Table 18:** Composition of flue gases during LLDPE combustion in a fluidised bed of Fe_2_O_3_-cenospheres at 500 °C.

Temp.	Polymer	Mass	Time	CO2	CO	CH4	C2H6	C3H8	C2H4	C4+	HCHO	CH3CHO	Acrolein	C2H2	C6H6	CH3OH	CH3COOH	CH3COCH3
°C	-	mg	s	%_vol._	ppm													
500	LLDPE	196	0	0.00	0.00	0.00	0.00	0.00	0.00	0.00	0.00	0.00	0.00	0.00	0.00	0.00	0.00	0.00
			7	0.08	37.82	0.61	0.00	0.00	0.96	1.03	0.33	0.00	0.00	0.00	0.00	0.00	0.00	0.00
			15	0.47	278.58	7.30	0.00	0.00	16.57	20.62	6.86	0.00	0.00	0.00	3.48	0.00	0.00	0.00
			22	1.25	802.21	16.09	0.00	0.00	71.69	107.77	39.50	29.15	0.00	5.45	20.64	0.00	0.00	0.00
			29	1.38	1201.44	27.83	0.00	0.00	113.50	205.03	81.58	123.53	0.00	13.98	35.37	0.00	0.00	0.00
			37	1.23	835.99	21.07	0.00	0.00	78.44	163.18	63.55	114.39	0.00	10.41	27.92	0.00	0.00	0.00
			44	0.45	185.24	17.06	0.00	0.00	12.17	57.40	11.90	8.30	0.00	2.22	10.66	0.00	0.00	0.00
			52	0.15	48.16	8.99	0.00	0.00	2.84	26.14	1.80	0.00	0.00	0.65	9.19	0.00	0.00	0.00
			59	0.06	15.43	5.11	0.00	0.00	0.00	14.29	0.00	0.00	0.00	0.00	2.24	0.00	0.00	0.00
			66	0.02	5.77	3.78	0.00	0.00	0.00	8.62	0.00	0.00	0.00	0.00	1.28	0.00	0.00	0.00
			73	0.01	2.10	2.33	0.00	0.00	0.00	6.12	0.00	0.00	0.00	0.00	1.31	0.00	0.00	0.00

		298	0	0.00	0.00	0.00	0.00	0.00	0.00	0.00	0.00	0.00	0.00	0.00	0.00	0.00	0.00	0.00
			7	0.12	53.97	2.63	0.00	0.00	2.17	0.77	0.11	0.00	0.00	0.00	1.36	0.00	0.00	0.00
			15	0.61	400.12	10.37	0.00	0.00	21.77	28.93	11.26	0.00	0.00	2.40	5.29	0.00	0.00	0.00
			22	1.33	1185.06	23.20	0.00	0.00	103.58	164.25	66.42	94.10	0.00	8.11	26.07	0.00	0.00	0.00
			29	1.77	1791.09	44.03	0.00	0.00	167.38	303.02	142.19	227.62	0.00	22.89	38.29	0.00	0.00	0.00
			36	1.77	1299.23	33.73	0.00	0.00	124.46	244.69	96.85	168.48	0.00	14.95	50.36	0.00	0.00	0.00
			43	1.01	412.57	18.33	0.00	0.00	30.13	105.94	25.82	67.33	0.00	5.39	19.25	0.00	0.00	0.00
			50	0.40	121.62	15.42	0.00	0.00	4.89	46.40	4.15	29.58	0.00	1.69	10.70	0.00	0.00	0.00
			58	0.17	39.56	7.77	0.00	0.00	0.87	24.91	0.00	0.00	0.00	0.00	7.46	0.00	0.00	0.00
			65	0.08	14.99	9.02	0.00	0.00	0.00	16.22	0.00	0.00	0.00	0.00	3.75	0.00	0.00	0.00
			72	0.04	7.39	3.54	0.00	0.00	0.00	11.25	0.00	0.00	0.00	0.00	2.93	0.00	0.00	0.00
			79	0.03	3.80	2.59	0.00	0.00	0.00	8.25	0.00	0.00	0.00	0.00	1.92	0.00	0.00	0.00

		96	0	0.00	0.00	0.00	0.00	0.00	0.00	0.00	0.00	0.00	0.00	0.00	0.00	0.00	0.00	0.00
			8	0.16	68.35	2.08	0.00	0.00	5.40	3.33	0.75	0.00	0.00	0.00	1.40	0.00	0.00	0.00
			15	0.46	248.18	7.79	0.00	0.00	15.44	18.24	5.95	0.00	0.00	1.75	3.71	0.00	0.00	0.00
			22	0.77	418.51	18.06	0.00	0.00	39.53	47.20	18.41	0.00	0.00	3.68	7.14	0.00	0.00	0.00
			29	1.05	557.91	11.30	0.00	0.00	54.73	87.07	38.16	35.92	0.00	6.00	15.83	0.00	0.00	0.00
			36	0.64	258.03	4.05	0.00	0.00	18.21	48.69	16.77	9.42	0.00	3.63	8.90	0.00	0.00	0.00
			43	0.21	62.08	5.90	0.00	0.00	4.07	16.83	3.37	7.70	0.00	0.66	2.73	0.00	0.00	0.00
			50	0.04	12.20	3.66	0.00	0.00	0.89	5.11	0.00	0.00	0.00	0.15	0.86	0.00	0.00	0.00
			58	0.01	0.40	0.80	0.00	0.00	0.00	1.47	0.00	0.00	0.00	0.00	0.77	0.00	0.00	0.00
65	0.00		0.00	0.39	0.00	0.00	0.00	0.00	0.00	0.00	0.00	0.00	0.00	0.00	0.00	0.00
72	0.00		0.00	0.00	0.00	0.00	0.00	0.00	0.00	0.00	0.00	0.00	0.00	0.00	0.00	0.00

**Table 19:** Composition of flue gases during HDPE combustion in a fluidised bed of Fe_2_O_3_-cenospheres at 500 °C.

Temp.	Polymer	Mass	Time	CO2	CO	CH4	C2H6	C3H8	C2H4	C4+	HCHO	CH3CHO	Acrolein	C2H2	C6H6	CH3OH	CH3COOH	CH3COCH3
°C	-	mg	s	%_vol._	ppm													
500	HDPE	199	0	0.00	0.00	0.00	0.00	0.00	0.00	0.00	0.00	0.00	0.00	0.00	0.00	0.00	0.00	0.00
			7	0.07	27.68	0.00	0.00	0.00	0.89	0.00	0.52	0.00	0.00	1.00	0.00	0.00	0.00	0.00
			14	0.55	315.24	8.50	0.00	0.00	20.69	21.47	10.33	0.00	0.00	2.57	5.14	0.00	0.00	0.00
			21	1.25	785.54	11.85	0.00	0.00	68.35	87.97	39.85	30.21	0.00	8.22	19.18	0.00	0.00	0.00
			29	1.49	1172.55	24.95	0.00	0.00	105.81	167.24	79.81	118.41	0.00	13.56	37.83	0.00	0.00	0.00
			36	1.21	844.52	16.86	0.00	0.00	77.84	144.46	58.74	101.10	0.00	10.69	30.81	0.00	0.00	0.00
			43	0.74	339.35	10.70	0.00	0.00	25.76	74.22	19.29	54.99	0.00	5.04	22.31	0.00	0.00	0.00
			50	0.27	91.84	8.86	0.00	0.00	3.30	29.81	4.12	34.56	0.00	0.67	6.31	0.00	0.00	0.00
			57	0.09	24.88	4.08	0.00	0.00	0.00	13.66	0.02	11.38	0.00	0.00	4.03	0.00	0.00	0.00
			64	0.03	6.76	1.58	0.00	0.00	0.77	5.99	0.00	7.55	0.00	0.00	0.00	0.00	0.00	0.00
			71	0.01	1.83	0.00	0.00	0.00	0.00	2.80	0.00	4.59	0.00	0.00	0.00	0.00	0.00	0.00

		297	0	0.00	0.00	0.00	0.00	0.00	0.00	0.00	0.00	0.00	0.00	0.00	0.00	0.00	0.00	0.00
			7	0.02	8.86	0.00	0.00	0.00	0.00	0.00	0.00	0.00	0.00	0.24	0.00	0.00	0.00	0.00
			14	0.23	125.69	1.39	0.00	0.00	5.17	3.25	1.91	0.00	0.00	0.61	0.97	0.00	0.00	0.00
			21	0.74	489.24	4.86	0.00	0.00	33.42	40.44	21.41	0.00	0.00	0.82	7.01	0.00	0.00	0.00
			28	1.39	1260.97	25.05	0.00	0.00	108.32	166.43	88.91	118.06	0.00	8.81	26.61	0.00	0.00	0.00
			36	1.81	1532.99	31.83	0.00	0.00	139.23	248.10	132.72	214.75	0.00	17.71	38.39	0.00	0.00	0.00
			43	1.53	1095.60	24.28	0.00	0.00	98.56	203.71	80.65	152.39	0.00	13.19	40.36	0.00	0.00	0.00
			50	1.01	560.81	19.78	0.00	0.00	40.79	129.24	41.32	97.52	0.00	7.92	35.47	0.00	0.00	0.00
			57	0.61	263.84	15.67	0.00	0.00	23.80	72.05	19.52	53.61	0.00	4.63	15.77	0.00	0.00	0.00
			64	0.24	71.85	12.19	0.00	0.00	4.77	35.26	2.96	24.83	0.00	2.38	2.31	0.00	0.00	0.00
			71	0.08	20.06	6.93	0.00	0.00	0.00	19.82	0.00	20.14	0.00	0.51	2.53	0.00	0.00	0.00
			78	0.02	7.32	2.99	0.00	0.00	0.00	13.21	0.00	12.37	0.00	0.00	0.00	0.00	0.00	0.00

		102	0	0.00	0.00	0.00	0.00	0.00	0.00	0.00	0.00	0.00	0.00	0.00	0.00	0.00	0.00	0.00
			7	0.03	3.90	0.00	0.00	0.00	0.52	0.00	0.00	0.00	0.00	0.00	0.00	0.00	0.00	0.00
			14	0.28	130.57	1.37	0.00	0.00	6.90	4.46	3.10	0.00	0.00	0.10	2.51	0.00	0.00	0.00
			21	0.77	489.23	0.85	0.00	0.00	40.70	49.99	22.42	1.51	0.00	1.81	12.36	0.00	0.00	0.00
			28	1.27	697.66	9.42	0.00	0.00	63.10	97.55	42.45	57.47	0.00	8.32	24.59	0.00	0.00	0.00
			35	0.67	278.88	6.78	0.00	0.00	19.11	46.80	15.42	26.67	0.00	4.63	7.66	0.00	0.00	0.00
			43	0.25	71.12	2.65	0.00	0.00	3.35	14.43	2.50	7.21	0.00	1.55	3.23	0.00	0.00	0.00
			50	0.07	17.42	0.00	0.00	0.00	0.00	2.89	0.00	1.09	0.00	0.39	0.00	0.00	0.00	0.00
57	0.02		4.04	0.00	0.00	0.00	0.00	0.00	0.00	0.00	0.00	0.00	0.00	0.00	0.00	0.00
64	0.01		0.00	0.00	0.00	0.00	0.00	0.00	0.00	0.00	0.00	0.00	0.00	0.00	0.00	0.00

**Table 20:** Composition of flue gases during PE combustion in a fluidised bed of Fe_2_O_3_-cenospheres at 600 °C.

Temp.	Polymer	Mass	Time	CO2	CO	CH4	C2H6	C3H8	C2H4	C4+	HCHO	CH3CHO	Acrolein	C2H2	C6H6	CH3OH	CH3COOH	CH3COCH3
°C	-	mg	s	%_vol._	ppm													
600	PE	196	0	0.00	0.00	0.00	0.00	0.00	0.00	0.00	0.00	0.00	0.00	0.00	0.00	0.00	0.00	0.00
			7	0.50	274.10	11.83	11.21	0.00	54.30	17.38	9.50	4.95	0.00	3.16	10.12	0.00	0.00	0.00
			14	1.99	1369.52	100.17	63.55	0.00	323.83	136.60	88.40	59.35	0.00	10.38	81.85	0.00	0.00	9.64
			21	1.51	1318.08	113.34	29.00	0.00	341.36	131.84	104.58	77.50	0.00	21.81	84.31	0.00	0.00	7.56
			29	0.74	354.13	33.99	1.31	0.00	110.31	48.26	29.17	18.13	0.00	3.02	26.13	0.00	0.00	1.19
			36	0.27	82.54	14.11	0.00	0.00	26.12	16.00	6.93	0.00	0.00	0.03	8.93	0.00	0.00	0.13
			43	0.09	19.78	5.09	0.00	0.00	7.41	6.88	1.70	0.00	0.00	0.00	3.50	0.00	0.00	0.00
			50	0.03	5.75	1.78	0.00	0.00	2.84	3.43	0.00	0.00	0.00	0.00	0.00	0.00	0.00	0.00
			57	0.00	0.00	0.00	0.00	0.00	0.00	0.00	0.00	0.00	0.00	0.00	0.00	0.00	0.00	0.00

		304	0	0.00	0.00	0.00	0.00	0.00	0.00	0.00	0.00	0.00	0.00	0.00	0.00	0.00	0.00	0.00
			7	0.11	35.23	1.02	0.60	0.00	6.45	1.15	0.15	0.00	0.00	0.00	0.72	0.00	0.00	0.00
			14	1.35	943.58	53.70	29.52	0.00	201.35	64.80	50.28	35.94	0.00	5.45	45.73	0.00	0.00	7.62
			21	3.63	2910.01	263.32	36.89	0.00	753.29	175.89	213.61	258.99	0.00	25.09	225.72	0.00	0.00	49.76
			28	1.67	1291.98	154.84	40.19	0.00	408.44	154.29	110.92	83.08	0.00	22.72	109.57	0.00	0.00	10.17
			36	0.77	326.38	44.33	3.64	0.00	126.40	54.54	27.59	0.00	0.00	6.38	30.86	0.00	0.00	7.80
			43	0.30	78.06	12.33	0.00	0.00	34.60	20.29	7.46	0.00	0.00	0.34	13.37	0.00	0.00	0.00
			50	0.11	20.17	8.24	0.00	0.00	8.60	9.94	1.09	0.00	0.00	0.00	4.07	0.00	0.00	0.00
			57	0.05	5.79	2.32	0.00	0.00	1.82	5.29	0.40	0.00	0.00	0.00	3.03	0.00	0.00	0.00
			64	0.03	1.49	1.38	0.00	0.00	1.38	3.75	0.00	0.00	0.00	0.00	1.31	0.00	0.00	0.00
			71	0.00	1.34	0.00	0.00	0.00	0.00	2.69	0.00	0.00	0.00	0.00	0.00	0.00	0.00	0.00

		100	0	0.00	0.00	0.00	0.00	0.00	0.00	0.00	0.00	0.00	0.00	0.00	0.00	0.00	0.00	0.00
			7	0.05	13.90	0.00	0.00	0.00	1.65	0.00	0.43	0.00	0.00	0.07	1.17	0.00	0.00	0.00
			14	0.68	320.47	13.48	8.70	0.00	61.97	17.45	9.26	0.00	0.00	4.64	10.65	0.00	0.00	0.00
			21	1.30	883.52	49.10	22.82	0.00	192.38	62.87	50.67	29.83	0.00	6.38	39.94	0.00	0.00	6.25
			28	0.85	418.95	27.84	8.57	0.00	106.87	40.67	28.59	16.59	0.00	3.50	23.45	0.00	0.00	1.30
			35	0.28	102.93	10.44	4.34	0.00	25.90	12.32	6.79	0.00	0.00	1.39	7.32	0.00	0.00	0.00
			43	0.10	25.52	2.38	0.00	0.00	5.04	3.70	0.00	0.00	0.00	0.00	3.23	0.00	0.00	0.00
			50	0.02	6.56	0.18	0.00	0.00	1.94	1.16	0.00	0.00	0.00	0.00	2.61	0.00	0.00	0.00
57	0.00		1.40	0.00	0.00	0.00	0.00	0.00	0.00	0.00	0.00	0.00	1.08	0.00	0.00	0.00
64	0.00		0.00	0.00	0.00	0.00	0.00	0.00	0.00	0.00	0.00	0.00	0.87	0.00	0.00	0.00

**Table 21:** Composition of flue gases during LLDPE combustion in a fluidised bed of Fe_2_O_3_-cenospheres at 600 °C.

Temp.	Polymer	Mass	Time	CO2	CO	CH4	C2H6	C3H8	C2H4	C4+	HCHO	CH3CHO	Acrolein	C2H2	C6H6	CH3OH	CH3COOH	CH3COCH3
°C	-	mg	s	%_vol._	ppm													
600	LLDPE	202	0	0.00	0.00	0.00	0.00	0.00	0.00	0.00	0.00	0.00	0.00	0.00	0.00	0.00	0.00	0.00
			7	0.03	4.20	0.00	0.00	0.00	0.00	0.00	0.26	2.04	0.00	0.63	0.33	0.00	0.00	0.00
			14	0.62	301.04	14.88	11.32	0.00	62.76	21.84	10.43	4.41	0.00	2.72	12.06	0.00	0.00	0.00
			21	2.30	1424.97	117.06	81.33	0.00	361.43	141.89	104.87	78.22	9.68	14.09	83.11	29.07	0.00	20.77
			28	1.53	1044.38	104.91	35.87	0.00	313.10	107.95	95.64	72.06	0.00	7.55	73.10	0.00	0.00	4.31
			35	0.59	265.82	28.74	4.97	0.00	94.80	37.16	23.79	15.53	0.00	2.08	20.17	0.00	0.00	0.47
			42	0.23	65.28	11.04	1.45	0.00	23.64	12.58	5.89	0.00	0.00	0.98	7.31	0.00	0.00	0.00
			50	0.08	16.11	3.78	0.00	0.00	4.23	5.39	1.34	0.00	0.00	0.19	2.65	0.00	0.00	0.00
			57	0.04	3.80	0.70	0.00	0.00	0.14	2.68	0.05	0.00	0.00	0.00	1.03	0.00	0.00	0.00
			64	0.02	1.22	1.00	0.00	0.00	0.00	1.42	0.00	0.00	0.00	0.00	0.00	0.00	0.00	0.00
			71	0.00	0.00	0.00	0.00	0.00	0.00	0.00	0.00	0.00	0.00	0.00	0.00	0.00	0.00	0.00

		302	0	0.00	0.00	0.00	0.00	0.00	0.00	0.00	0.00	0.00	0.00	0.00	0.00	0.00	0.00	0.00
			8	0.08	31.72	0.64	0.00	0.00	4.36	0.89	1.17	0.00	0.00	0.05	0.84	0.00	0.00	0.00
			15	1.40	1316.15	84.36	44.68	14.82	298.23	92.76	80.07	58.83	12.46	10.49	74.62	0.00	0.00	13.10
			22	3.01	2781.98	233.96	56.39	29.80	733.85	242.70	213.72	178.05	0.11	22.44	156.99	0.00	0.00	26.73
			29	1.39	1209.45	138.27	44.75	0.00	384.69	129.94	108.05	79.76	0.00	20.25	95.43	0.00	0.00	7.75
			36	0.63	298.21	38.38	5.73	0.00	120.67	45.89	27.60	3.12	0.00	2.99	28.15	0.00	0.00	1.25
			43	0.22	72.89	14.84	0.49	0.00	28.71	15.72	7.00	0.00	0.00	0.00	10.32	0.00	0.00	0.09
			50	0.06	18.72	5.55	0.00	0.00	4.57	6.96	0.00	0.00	0.00	0.00	5.03	0.00	0.00	0.00
			58	0.02	5.50	1.70	0.00	0.00	0.49	3.71	0.00	0.00	0.00	0.00	1.62	0.00	0.00	0.00
			65	0.01	0.91	0.96	0.00	0.00	0.00	2.37	0.00	0.00	0.00	0.00	0.00	0.00	0.00	0.00

		102	0	0.00	0.00	0.00	0.00	0.00	0.00	0.00	0.00	0.00	0.00	0.00	0.00	0.00	0.00	0.00
			7	0.03	7.17	1.68	0.00	0.00	0.71	0.15	0.00	0.00	0.00	0.10	0.33	0.00	0.00	0.00
			15	0.54	249.13	11.74	8.92	0.00	50.38	16.22	7.04	0.00	0.00	2.42	8.73	0.00	0.00	0.00
			22	1.30	683.19	40.09	21.14	0.00	153.71	56.07	34.83	15.99	0.00	4.99	29.54	0.00	0.00	4.16
			29	0.85	374.84	26.97	9.88	0.00	92.73	37.59	22.21	10.01	0.00	2.41	18.61	0.00	0.00	0.82
			36	0.32	94.52	11.00	6.14	0.00	21.66	12.18	5.16	0.00	0.00	0.66	5.60	0.00	0.00	0.00
			43	0.12	23.42	4.13	0.00	0.00	5.27	3.95	0.00	0.00	0.00	0.00	2.82	0.00	0.00	0.00
			50	0.05	6.35	1.17	0.00	0.00	0.32	1.14	1.02	0.00	0.00	0.00	0.72	0.00	0.00	0.00
58	0.02		2.00	0.49	0.00	0.00	1.52	0.35	0.00	0.00	0.00	0.00	0.00	0.00	0.00	0.00
65	0.00		0.50	0.00	0.00	0.00	0.59	0.00	0.00	0.00	0.00	0.00	0.00	0.00	0.00	0.00

**Table 22:** Composition of flue gases during HDPE combustion in a fluidised bed of Fe_2_O_3_-cenospheres at 600 °C.

Temp.	Polymer	Mass	Time	CO2	CO	CH4	C2H6	C3H8	C2H4	C4+	HCHO	CH3CHO	Acrolein	C2H2	C6H6	CH3OH	CH3COOH	CH3COCH3
°C	-	mg	s	%_vol._	ppm													
600	HDPE	203	0	0.00	0.00	0.00	0.00	0.00	0.00	0.00	0.00	0.00	0.00	0.00	0.00	0.00	0.00	0.00
			7	0.32	145.70	10.75	10.39	0.00	39.49	11.73	10.70	2.63	0.00	0.00	7.79	0.00	0.00	0.00
			14	2.10	1952.25	149.04	57.95	0.00	475.65	180.71	146.35	121.58	0.00	0.00	125.43	0.00	5.04	15.64
			22	1.56	1312.08	127.33	32.55	0.00	364.42	151.64	108.57	101.32	0.00	24.69	99.16	0.00	2.79	9.32
			29	0.76	363.19	37.05	0.34	0.00	119.75	56.68	31.40	0.00	0.00	3.31	29.38	0.00	0.00	2.00
			36	0.26	88.57	17.03	0.00	0.00	32.86	20.26	7.58	0.00	0.00	0.54	9.19	0.00	0.00	0.00
			43	0.09	22.94	4.26	0.00	0.00	8.39	8.91	1.83	0.00	0.00	0.44	4.12	0.00	0.00	0.00
			50	0.04	5.73	1.32	0.00	0.00	1.12	4.83	0.00	2.00	0.00	0.05	2.76	0.00	0.00	0.00
			57	0.01	2.11	1.67	0.00	0.00	0.00	3.13	0.00	0.00	0.00	0.00	0.46	0.00	0.00	0.00
			66	0.00	0.21	1.22	0.00	0.00	0.00	1.93	0.00	0.00	0.00	0.00	0.66	0.00	0.00	0.00
			73	0.00	0.00	0.00	0.00	0.00	0.00	0.00	0.00	0.00	0.00	0.00	0.13	0.00	0.00	0.00

		301	0	0.00	0.00	0.00	0.00	0.00	0.00	0.00	0.00	0.00	0.00	0.00	0.00	0.00	0.00	0.00
			7	0.05	15.26	0.00	0.00	0.00	1.36	0.23	0.51	0.00	0.00	0.00	0.38	0.00	0.00	0.00
			14	0.70	384.44	16.51	10.60	0.00	76.49	25.10	16.06	6.02	0.00	0.00	13.87	0.00	0.00	0.00
			22	1.83	1845.94	140.72	52.80	0.00	444.65	169.40	133.00	103.71	0.00	0.00	114.82	0.00	0.00	14.11
			29	1.96	1810.27	162.69	48.70	0.00	481.15	183.87	141.04	83.67	0.00	0.00	126.18	0.00	0.00	14.97
			36	1.06	555.71	58.97	7.43	0.00	183.03	74.38	48.52	9.03	0.00	0.00	45.10	0.00	0.00	0.26
			43	0.35	133.57	15.33	0.00	0.00	49.88	26.45	11.78	2.94	0.00	0.00	16.68	0.00	0.00	0.00
			50	0.12	34.65	8.06	0.00	0.00	10.23	10.45	2.50	0.00	0.00	0.00	6.57	0.00	0.00	0.00
			57	0.04	8.73	3.36	0.00	0.00	1.65	5.34	0.47	0.00	0.00	0.00	3.64	0.00	0.00	0.00
			65	0.01	2.09	1.68	0.00	0.00	1.73	3.34	0.00	0.00	0.00	0.00	2.19	0.00	0.00	0.00
			72	0.00	0.98	0.00	0.00	0.00	0.00	0.00	0.00	0.00	0.00	0.00	0.00	0.00	0.00	0.00

		101	0	0.00	0.00	0.00	0.00	0.00	0.00	0.00	0.00	0.00	0.00	0.00	0.00	0.00	0.00	0.00
			7	0.09	28.98	1.56	0.00	0.00	0.70	1.23	1.21	0.00	0.00	0.62	1.67	0.00	0.00	0.00
			14	0.89	442.69	23.54	11.76	0.00	93.38	32.83	20.71	0.00	0.00	4.59	18.77	0.00	0.00	0.67
			22	1.22	605.05	42.96	16.58	0.00	141.02	62.96	32.56	0.00	0.00	4.99	31.86	0.00	0.00	3.78
			29	0.93	392.91	29.09	5.22	0.00	93.34	44.14	21.62	0.00	0.00	2.18	21.61	0.00	0.00	1.07
			36	0.35	107.82	13.80	0.00	0.00	25.80	15.33	5.85	0.00	0.00	0.99	8.69	0.00	0.00	0.00
			43	0.12	27.48	4.47	0.00	0.00	5.66	5.39	1.48	0.00	0.00	0.91	2.84	0.00	0.00	0.00
			50	0.05	6.93	1.31	0.00	0.00	2.06	2.16	0.00	0.00	0.00	0.68	0.64	0.00	0.00	0.00
			57	0.01	2.00	0.72	0.00	0.00	0.12	0.73	0.00	0.00	0.00	0.00	0.00	0.00	0.00	0.00
64	0.00		0.99	0.67	0.00	0.00	1.25	0.13	0.00	0.00	0.00	0.00	0.00	0.00	0.00	0.00
72	0.00		0.23	0.19	0.00	0.00	0.00	0.00	0.00	0.00	0.00	0.00	0.00	0.00	0.00	0.00

**Table 23:** Composition of flue gases during PE combustion in a fluidised bed of Fe_2_O_3_-cenospheres at 700 °C.

Temp.	Polymer	Mass	Time	CO2	CO	CH4	C2H6	C3H8	C2H4	C4+	HCHO	CH3CHO	Acrolein	C2H2	C6H6	CH3OH	CH3COOH	CH3COCH3
°C	-	mg	s	%_vol._	ppm													
700	PE	198	0	0.00	0.00	0.00	0.00	0.00	0.00	0.00	0.00	0.00	0.00	0.00	0.00	0.00	0.00	0.00
			7	0.03	10.97	0.71	0.00	0.00	0.68	0.00	0.00	0.00	0.00	0.00	0.33	0.00	0.00	0.00
			14	1.16	810.84	18.32	0.00	0.00	18.95	0.00	4.60	0.00	0.00	0.00	2.23	0.00	0.00	0.00
			22	2.83	1210.56	29.90	0.00	0.00	25.11	0.00	6.32	0.00	0.00	0.00	1.61	0.00	0.00	0.00
			29	1.35	523.57	18.13	0.00	0.00	13.20	0.00	2.46	0.00	0.00	0.00	1.55	0.00	0.00	0.00
			36	0.43	121.34	4.45	0.00	0.00	2.21	0.00	0.00	0.00	0.00	0.00	1.30	0.00	0.00	0.00
			43	0.14	28.68	0.99	0.00	0.00	0.00	0.00	0.00	0.00	0.00	0.00	0.52	0.00	0.00	0.00
			50	0.05	6.66	0.28	0.00	0.00	0.00	0.00	0.00	0.00	0.00	0.00	0.33	0.00	0.00	0.00
			57	0.02	1.39	0.03	0.00	0.00	0.00	0.00	0.00	0.00	0.00	0.00	0.00	0.00	0.00	0.00
			64	0.00	0.00	0.00	0.00	0.00	0.00	0.00	0.00	0.00	0.00	0.00	0.00	0.00	0.00	0.00

		205	0	0.00	0.00	0.00	0.00	0.00	0.00	0.00	0.00	0.00	0.00	0.00	0.00	0.00	0.00	0.00
			7	0.03	3.78	0.05	0.00	0.00	0.30	0.00	0.00	0.00	0.00	0.00	0.00	0.00	0.00	0.00
			15	1.32	699.83	12.63	0.00	0.00	11.09	0.00	1.98	0.00	0.00	0.00	0.00	0.00	0.00	0.00
			22	3.15	910.71	16.86	0.00	0.00	14.10	0.00	4.23	0.00	0.00	0.00	0.00	0.00	0.00	0.00
			29	1.38	421.52	10.20	0.00	0.00	6.34	0.00	1.29	0.00	0.00	0.00	0.00	0.00	0.00	0.00
			36	0.44	98.16	1.96	0.00	0.00	1.75	0.00	0.58	0.00	0.00	0.00	0.00	0.00	0.00	0.00
			43	0.13	25.33	0.14	0.00	0.00	0.05	0.00	0.00	0.00	0.00	0.00	0.00	0.00	0.00	0.00
			50	0.05	7.01	0.00	0.00	0.00	0.00	0.00	0.00	0.00	0.00	0.00	0.00	0.00	0.00	0.00
			58	0.01	2.20	0.00	0.00	0.00	0.00	0.00	0.00	0.00	0.00	0.00	0.00	0.00	0.00	0.00
			65	0.00	0.00	0.00	0.00	0.00	0.00	0.00	0.00	0.00	0.00	0.00	0.00	0.00	0.00	0.00

		301	0	0.00	0.00	0.00	0.00	0.00	0.00	0.00	0.00	0.00	0.00	0.00	0.00	0.00	0.00	0.00
			7	0.13	70.69	3.29	0.00	0.00	4.77	0.00	1.35	0.00	0.00	0.81	0.02	0.00	0.00	0.00
			14	2.94	1022.40	48.67	3.69	0.00	67.46	0.00	10.19	0.00	0.00	3.81	4.03	0.00	0.00	0.00
			22	3.84	1597.75	82.35	5.52	0.00	106.05	0.00	14.25	0.00	0.00	6.01	7.52	0.00	0.00	0.00
			29	1.46	487.02	23.61	0.57	0.00	29.74	0.00	3.59	0.00	0.00	0.59	1.48	0.00	0.00	0.00
			36	0.45	113.05	5.24	0.00	0.00	5.56	0.00	0.64	0.00	0.00	0.00	0.49	0.00	0.00	0.00
			43	0.14	27.72	0.90	0.00	0.00	0.27	0.00	0.00	0.00	0.00	0.00	0.00	0.00	0.00	0.00
			50	0.05	7.13	0.00	0.00	0.00	0.00	0.00	0.00	0.00	0.00	0.00	0.00	0.00	0.00	0.00
			57	0.02	2.16	0.00	0.00	0.00	0.00	0.00	0.00	0.00	0.00	0.00	0.00	0.00	0.00	0.00
64	0.02		1.22	0.00	0.00	0.00	0.00	0.00	0.00	0.00	0.00	0.00	0.00	0.00	0.00	0.00
72	0.00		0.00	0.00	0.00	0.00	0.00	0.00	0.00	0.00	0.00	0.00	0.00	0.00	0.00	0.00

**Table 24:** Composition of flue gases during LLDPE combustion in a fluidised bed of Fe_2_O_3_-cenospheres at 700 °C.

Temp.	Polymer	Mass	Time	CO2	CO	CH4	C2H6	C3H8	C2H4	C4+	HCHO	CH3CHO	Acrolein	C2H2	C6H6	CH3OH	CH3COOH	CH3COCH3
°C	-	mg	s	%_vol._	ppm													
700	LLDPE	199	0	0.00	0.00	0.00	0.00	0.00	0.00	0.00	0.00	0.00	0.00	0.00	0.00	0.00	0.00	0.00
			7	0.17	112.04	5.52	0.00	0.00	10.56	0.00	1.39	0.35	0.00	0.00	1.00	0.00	0.00	0.00
			14	2.22	1107.80	26.85	0.00	0.00	33.80	0.00	7.20	0.44	0.00	0.00	2.20	0.00	0.00	0.00
			22	2.17	859.21	28.34	0.00	0.00	25.33	0.00	5.76	2.53	0.00	0.00	2.08	0.00	0.00	0.00
			29	0.91	225.63	8.64	0.00	0.00	6.36	0.00	1.54	1.05	0.00	0.00	1.17	0.00	0.00	0.00
			36	0.31	52.52	2.48	0.00	0.00	0.38	0.00	0.17	0.91	0.00	0.00	0.78	0.00	0.00	0.00
			43	0.08	12.20	0.12	0.00	0.00	0.00	0.00	0.00	0.00	0.00	0.00	0.00	0.00	0.00	0.00
			50	0.02	3.24	0.00	0.00	0.00	0.00	0.00	0.00	0.00	0.00	0.00	0.00	0.00	0.00	0.00
			57	0.02	0.78	0.00	0.00	0.00	0.00	0.00	0.00	0.00	0.00	0.00	0.00	0.00	0.00	0.00
			64	0.00	0.00	0.00	0.00	0.00	0.00	0.00	0.00	0.00	0.00	0.00	0.00	0.00	0.00	0.00

		298	0	0.00	0.00	0.00	0.00	0.00	0.00	0.00	0.00	0.00	0.00	0.00	0.00	0.00	0.00	0.00
			7	0.20	116.10	6.35	0.00	0.00	12.43	0.00	1.53	0.00	0.00	1.08	1.49	0.00	0.00	0.00
			14	3.21	1025.79	35.86	0.00	0.00	51.49	0.00	8.28	0.15	0.00	6.84	3.19	0.00	0.00	0.00
			21	3.86	875.75	38.16	0.00	0.00	43.99	0.00	7.98	1.76	0.00	9.45	4.08	0.00	0.00	0.00
			28	1.47	243.11	11.80	0.00	0.00	12.48	0.00	1.93	0.93	0.00	2.15	1.98	0.00	0.00	0.00
			36	0.50	56.60	2.81	0.00	0.00	2.33	0.00	0.00	0.03	0.00	1.65	0.97	0.00	0.00	0.00
			43	0.17	13.77	0.87	0.00	0.00	0.24	0.00	0.00	0.00	0.00	0.62	0.89	0.00	0.00	0.00
			50	0.06	3.42	0.06	0.00	0.00	0.00	0.00	0.00	0.00	0.00	0.00	0.00	0.00	0.00	0.00
			57	0.02	0.00	0.00	0.00	0.00	0.00	0.00	0.00	0.00	0.00	0.00	0.00	0.00	0.00	0.00
			64	0.00	0.00	0.00	0.00	0.00	0.00	0.00	0.00	0.00	0.00	0.00	0.00	0.00	0.00	0.00

		100	0	0.00	0.00	0.00	0.00	0.00	0.00	0.00	0.00	0.00	0.00	0.00	0.00	0.00	0.00	0.00
			7	0.28	200.31	8.10	0.00	0.00	13.05	0.00	2.17	0.22	0.00	0.48	0.83	0.00	0.00	0.00
			14	1.20	1537.00	42.62	0.00	0.00	34.95	0.00	8.71	1.27	0.00	1.89	2.77	0.00	0.00	0.00
			21	0.97	1089.95	30.89	0.00	0.00	21.52	0.00	5.10	0.00	0.00	1.77	1.31	0.00	0.00	0.00
			28	0.41	277.27	8.25	0.00	0.00	3.35	0.00	0.56	0.00	0.00	0.00	0.00	0.00	0.00	0.00
			36	0.14	65.33	2.24	0.00	0.00	1.15	0.00	0.00	0.00	0.00	0.00	0.00	0.00	0.00	0.00
			43	0.02	16.45	0.26	0.00	0.00	0.99	0.00	0.00	0.00	0.00	0.00	0.00	0.00	0.00	0.00
50	0.00		4.26	0.00	0.00	0.00	0.00	0.00	0.00	0.00	0.00	0.00	0.00	0.00	0.00	0.00
57	0.00		1.12	0.00	0.00	0.00	0.00	0.00	0.00	0.00	0.00	0.00	0.00	0.00	0.00	0.00

**Table 25:** Composition of flue gases during HDPE combustion in a fluidised bed of Fe_2_O_3_-cenospheres at 700 °C.

Temp.	Polymer	Mass	Time	CO2	CO	CH4	C2H6	C3H8	C2H4	C4+	HCHO	CH3CHO	Acrolein	C2H2	C6H6	CH3OH	CH3COOH	CH3COCH3
°C	-	mg	s	%_vol._	ppm													
700	HDPE	201	0	0.00	0.00	0.00	0.00	0.00	0.00	0.00	0.00	0.00	0.00	0.00	0.00	0.00	0.00	0.00
			7	0.31	170.11	5.46	0.00	0.00	6.67	0.00	1.12	0.00	0.00	0.00	0.00	0.00	0.00	0.00
			14	2.10	1122.76	24.66	0.00	0.00	27.70	0.00	5.65	0.00	0.00	0.00	0.00	0.00	0.00	0.00
			21	1.89	1565.67	39.37	0.00	0.00	35.80	0.00	8.51	0.00	0.00	0.00	0.00	0.00	0.00	0.00
			28	0.82	484.81	14.41	0.00	0.00	16.82	0.00	2.50	0.00	0.00	0.00	0.00	0.00	0.00	0.00
			35	0.26	116.78	3.18	0.00	0.00	4.16	0.00	0.90	0.00	0.00	0.00	0.00	0.00	0.00	0.00
			43	0.09	28.67	1.29	0.00	0.00	1.41	0.00	0.00	0.00	0.00	0.00	0.00	0.00	0.00	0.00
			50	0.03	6.40	0.00	0.00	0.00	0.00	0.00	0.00	0.00	0.00	0.00	0.00	0.00	0.00	0.00
			57	0.02	0.00	0.00	0.00	0.00	0.00	0.00	0.00	0.00	0.00	0.00	0.00	0.00	0.00	0.00

		298	0	0.00	0.00	0.00	0.00	0.00	0.00	0.00	0.00	0.00	0.00	0.00	0.00	0.00	0.00	0.00
			8	0.08	33.85	1.61	0.00	0.00	1.51	0.00	0.00	0.00	0.00	0.00	0.00	0.00	0.00	0.00
			15	2.24	493.60	10.21	0.00	0.00	12.01	0.00	3.18	0.00	0.00	0.00	0.00	0.00	0.00	0.00
			22	4.37	775.24	17.84	0.00	0.00	15.23	0.00	3.74	0.00	0.00	0.00	0.00	0.00	0.00	0.00
			29	1.81	355.39	11.57	0.00	0.00	6.19	0.00	1.76	0.00	0.00	0.00	0.00	0.00	0.00	0.00
			36	0.59	82.94	2.91	0.00	0.00	1.87	0.00	0.10	0.00	0.00	0.00	0.00	0.00	0.00	0.00
			43	0.17	19.65	0.52	0.00	0.00	0.00	0.00	0.00	0.00	0.00	0.00	0.00	0.00	0.00	0.00
			51	0.06	4.92	0.30	0.00	0.00	0.00	0.00	0.00	0.00	0.00	0.00	0.00	0.00	0.00	0.00
			58	0.03	2.00	0.00	0.00	0.00	0.00	0.00	0.00	0.00	0.00	0.00	0.00	0.00	0.00	0.00

		99	0	0.00	0.00	0.00	0.00	0.00	0.00	0.00	0.00	0.00	0.00	0.00	0.00	0.00	0.00	0.00
			8	0.17	99.73	4.87	0.00	0.00	6.65	0.00	0.84	0.00	0.00	0.00	0.00	0.00	0.00	0.00
			15	1.47	1218.40	29.90	0.00	0.00	27.27	0.00	6.59	0.00	0.00	0.00	0.00	0.00	0.00	0.00
			22	1.00	907.99	30.08	0.00	0.00	39.60	0.00	8.68	0.00	0.00	0.00	0.00	0.00	0.00	0.00
			29	0.40	255.18	8.55	0.00	0.00	10.86	0.00	2.67	0.00	0.00	0.00	0.00	0.00	0.00	0.00
			36	0.12	60.61	1.86	0.00	0.00	3.79	0.00	0.00	0.00	0.00	0.00	0.00	0.00	0.00	0.00
			43	0.02	15.22	0.28	0.00	0.00	0.08	0.00	0.00	0.00	0.00	0.00	0.00	0.00	0.00	0.00
51	0.00		4.44	0.00	0.00	0.00	0.00	0.00	0.00	0.00	0.00	0.00	0.00	0.00	0.00	0.00
58	0.00		1.27	0.00	0.00	0.00	0.00	0.00	0.00	0.00	0.00	0.00	0.00	0.00	0.00	0.00

**Table 26:** Composition of flue gases during PE combustion in a fluidised bed of Fe_2_O_3_-cenospheres at 800 °C.

Temp.	Polymer	Mass	Time	CO2	CO	CH4	C2H6	C3H8	C2H4	C4+	HCHO	CH3CHO	Acrolein	C2H2	C6H6	CH3OH	CH3COOH	CH3COCH3
°C	-	mg	s	%_vol._	ppm													
800	PE	205	0	0.00	0.00	0.00	0.00	0.00	0.00	0.00	0.00	0.00	0.00	0.00	0.00	0.00	0.00	0.00
			7	1.83	45.28	1.93	0.00	0.00	0.00	0.00	0.00	0.00	0.00	0.00	0.00	0.00	0.00	0.00
			14	3.25	102.79	5.17	0.00	0.00	0.00	0.00	0.00	0.00	0.00	0.00	0.00	0.00	0.00	0.00
			21	1.09	25.98	1.62	0.00	0.00	0.00	0.00	0.00	0.00	0.00	0.00	0.00	0.00	0.00	0.00
			28	0.34	5.28	0.00	0.00	0.00	0.00	0.00	0.00	0.00	0.00	0.00	0.00	0.00	0.00	0.00
			36	0.10	1.37	0.00	0.00	0.00	0.00	0.00	0.00	0.00	0.00	0.00	0.00	0.00	0.00	0.00
			43	0.01	0.00	0.00	0.00	0.00	0.00	0.00	0.00	0.00	0.00	0.00	0.00	0.00	0.00	0.00
			50	0.00	0.00	0.00	0.00	0.00	0.00	0.00	0.00	0.00	0.00	0.00	0.00	0.00	0.00	0.00

		198	0	0.00	0.00	0.00	0.00	0.00	0.00	0.00	0.00	0.00	0.00	0.00	0.00	0.00	0.00	0.00
			7	0.12	2.24	0.00	0.00	0.00	0.00	0.00	0.00	0.00	0.00	0.00	0.00	0.00	0.00	0.00
			14	3.61	51.74	0.00	0.00	0.00	0.00	0.00	0.00	0.00	0.00	0.00	0.00	0.00	0.00	0.00
			21	2.19	21.94	0.00	0.00	0.00	0.00	0.00	0.00	0.00	0.00	0.00	0.00	0.00	0.00	0.00
			28	0.68	5.42	0.00	0.00	0.00	0.00	0.00	0.00	0.00	0.00	0.00	0.00	0.00	0.00	0.00
			36	0.23	0.87	0.00	0.00	0.00	0.00	0.00	0.00	0.00	0.00	0.00	0.00	0.00	0.00	0.00
			43	0.05	0.00	0.00	0.00	0.00	0.00	0.00	0.00	0.00	0.00	0.00	0.00	0.00	0.00	0.00
			50	0.03	0.00	0.00	0.00	0.00	0.00	0.00	0.00	0.00	0.00	0.00	0.00	0.00	0.00	0.00

		96	0	0.00	0.00	0.00	0.00	0.00	0.00	0.00	0.00	0.00	0.00	0.00	0.00	0.00	0.00	0.00
			7	0.41	3.57	0.00	0.00	0.00	0.00	0.00	0.00	0.00	0.00	0.00	0.00	0.00	0.00	0.00
			14	2.28	17.84	0.00	0.00	0.00	0.00	0.00	0.00	0.00	0.00	0.00	0.00	0.00	0.00	0.00
			21	0.94	6.06	0.00	0.00	0.00	0.00	0.00	0.00	0.00	0.00	0.00	0.00	0.00	0.00	0.00
			28	0.30	1.00	0.00	0.00	0.00	0.00	0.00	0.00	0.00	0.00	0.00	0.00	0.00	0.00	0.00
36	0.06		0.25	0.00	0.00	0.00	0.00	0.00	0.00	0.00	0.00	0.00	0.00	0.00	0.00	0.00
43	0.01		0.00	0.00	0.00	0.00	0.00	0.00	0.00	0.00	0.00	0.00	0.00	0.00	0.00	0.00

**Table 27:** Composition of flue gases during LLDPE combustion in a fluidised bed of Fe_2_O_3_-cenospheres at 800 °C.

Temp.	Polymer	Mass	Time	CO2	CO	CH4	C2H6	C3H8	C2H4	C4+	HCHO	CH3CHO	Acrolein	C2H2	C6H6	CH3OH	CH3COOH	CH3COCH3
°C	-	mg	s	%_vol._	ppm													
800	LLDPE	203	0	0.00	0.00	0.00	0.00	0.00	0.00	0.00	0.00	0.00	0.00	0.00	0.00	0.00	0.00	0.00
			7	0.22	3.22	0.00	0.00	0.00	0.00	0.00	0.00	0.00	0.00	0.00	0.00	0.00	0.00	0.00
			14	4.07	39.07	0.00	0.00	0.00	0.00	0.00	0.00	0.00	0.00	0.00	0.00	0.00	0.00	0.00
			21	2.05	21.70	0.00	0.00	0.00	0.00	0.00	0.00	0.00	0.00	0.00	0.00	0.00	0.00	0.00
			28	0.78	3.82	0.00	0.00	0.00	0.00	0.00	0.00	0.00	0.00	0.00	0.00	0.00	0.00	0.00
			35	0.23	0.92	0.00	0.00	0.00	0.00	0.00	0.00	0.00	0.00	0.00	0.00	0.00	0.00	0.00
			43	0.07	0.00	0.00	0.00	0.00	0.00	0.00	0.00	0.00	0.00	0.00	0.00	0.00	0.00	0.00
			50	0.01	0.00	0.00	0.00	0.00	0.00	0.00	0.00	0.00	0.00	0.00	0.00	0.00	0.00	0.00

		203	0	0.00	0.00	0.00	0.00	0.00	0.00	0.00	0.00	0.00	0.00	0.00	0.00	0.00	0.00	0.00
			7	0.87	20.37	0.00	0.00	0.00	0.00	0.00	0.00	0.00	0.00	0.00	0.00	0.00	0.00	0.00
			14	3.77	42.99	0.00	0.00	0.00	0.00	0.00	0.00	0.00	0.00	0.00	0.00	0.00	0.00	0.00
			21	1.57	14.17	0.00	0.00	0.00	0.00	0.00	0.00	0.00	0.00	0.00	0.00	0.00	0.00	0.00
			28	0.50	3.92	0.00	0.00	0.00	0.00	0.00	0.00	0.00	0.00	0.00	0.00	0.00	0.00	0.00
			35	0.17	0.85	0.00	0.00	0.00	0.00	0.00	0.00	0.00	0.00	0.00	0.00	0.00	0.00	0.00
			43	0.03	0.05	0.00	0.00	0.00	0.00	0.00	0.00	0.00	0.00	0.00	0.00	0.00	0.00	0.00
			50	0.02	0.00	0.00	0.00	0.00	0.00	0.00	0.00	0.00	0.00	0.00	0.00	0.00	0.00	0.00

		101	0	0.00	0.00	0.00	0.00	0.00	0.00	0.00	0.00	0.00	0.00	0.00	0.00	0.00	0.00	0.00
			7	0.32	2.47	0.00	0.00	0.00	0.00	0.00	0.00	0.00	0.00	0.00	0.00	0.00	0.00	0.00
			14	2.22	10.35	0.00	0.00	0.00	0.00	0.00	0.00	0.00	0.00	0.00	0.00	0.00	0.00	0.00
			21	1.11	3.45	0.00	0.00	0.00	0.00	0.00	0.00	0.00	0.00	0.00	0.00	0.00	0.00	0.00
			28	0.37	0.00	0.00	0.00	0.00	0.00	0.00	0.00	0.00	0.00	0.00	0.00	0.00	0.00	0.00
			35	0.10	0.00	0.00	0.00	0.00	0.00	0.00	0.00	0.00	0.00	0.00	0.00	0.00	0.00	0.00
			42	0.03	0.00	0.00	0.00	0.00	0.00	0.00	0.00	0.00	0.00	0.00	0.00	0.00	0.00	0.00
50	0.01		0.00	0.00	0.00	0.00	0.00	0.00	0.00	0.00	0.00	0.00	0.00	0.00	0.00	0.00
57	0.01		0.00	0.00	0.00	0.00	0.00	0.00	0.00	0.00	0.00	0.00	0.00	0.00	0.00	0.00

**Table 28:** Composition of flue gases during HDPE combustion in a fluidised bed of Fe_2_O_3_-cenospheres at 800 °C.

Temp.	Polymer	Mass	Time	CO2	CO	CH4	C2H6	C3H8	C2H4	C4+	HCHO	CH3CHO	Acrolein	C2H2	C6H6	CH3OH	CH3COOH	CH3COCH3
°C	-	mg	s	%_vol._	ppm													
800	HDPE	203	0	0.00	0.00	0.00	0.00	0.00	0.00	0.00	0.00	0.00	0.00	0.00	0.00	0.00	0.00	0.00
			8	0.10	0.99	0.00	0.00	0.00	0.00	0.00	0.00	0.00	0.00	0.00	0.00	0.00	0.00	0.00
			15	2.01	22.01	0.00	0.00	0.00	0.00	0.00	0.00	0.00	0.00	0.00	0.00	0.00	0.00	0.00
			22	2.96	23.12	0.00	0.00	0.00	0.00	0.00	0.00	0.00	0.00	0.00	0.00	0.00	0.00	0.00
			29	1.51	8.70	0.00	0.00	0.00	0.00	0.00	0.00	0.00	0.00	0.00	0.00	0.00	0.00	0.00
			36	0.48	2.56	0.00	0.00	0.00	0.00	0.00	0.00	0.00	0.00	0.00	0.00	0.00	0.00	0.00
			43	0.15	0.42	0.00	0.00	0.00	0.00	0.00	0.00	0.00	0.00	0.00	0.00	0.00	0.00	0.00
			50	0.05	0.05	0.00	0.00	0.00	0.00	0.00	0.00	0.00	0.00	0.00	0.00	0.00	0.00	0.00
			58	0.02	0.10	0.00	0.00	0.00	0.00	0.00	0.00	0.00	0.00	0.00	0.00	0.00	0.00	0.00

		199	0	0.00	0.00	0.00	0.00	0.00	0.00	0.00	0.00	0.00	0.00	0.00	0.00	0.00	0.00	0.00
			7	0.43	2.88	0.00	0.00	0.00	0.00	0.00	0.00	0.00	0.00	0.00	0.00	0.00	0.00	0.00
			15	4.05	30.77	0.00	0.00	0.00	0.00	0.00	0.00	0.00	0.00	0.00	0.00	0.00	0.00	0.00
			22	1.92	13.77	0.00	0.00	0.00	0.00	0.00	0.00	0.00	0.00	0.00	0.00	0.00	0.00	0.00
			29	0.61	3.12	0.00	0.00	0.00	0.00	0.00	0.00	0.00	0.00	0.00	0.00	0.00	0.00	0.00
			36	0.19	0.45	0.00	0.00	0.00	0.00	0.00	0.00	0.00	0.00	0.00	0.00	0.00	0.00	0.00
			43	0.04	0.00	0.00	0.00	0.00	0.00	0.00	0.00	0.00	0.00	0.00	0.00	0.00	0.00	0.00
			50	0.02	0.00	0.00	0.00	0.00	0.00	0.00	0.00	0.00	0.00	0.00	0.00	0.00	0.00	0.00
			57	0.00	0.00	0.00	0.00	0.00	0.00	0.00	0.00	0.00	0.00	0.00	0.00	0.00	0.00	0.00

		97	0	0.00	0.00	0.00	0.00	0.00	0.00	0.00	0.00	0.00	0.00	0.00	0.00	0.00	0.00	0.00
			7	0.79	5.98	0.00	0.00	0.00	0.00	0.00	0.00	0.00	0.00	0.00	0.00	0.00	0.00	0.00
			15	1.70	9.84	0.00	0.00	0.00	0.00	0.00	0.00	0.00	0.00	0.00	0.00	0.00	0.00	0.00
			22	0.89	5.10	0.00	0.00	0.00	0.00	0.00	0.00	0.00	0.00	0.00	0.00	0.00	0.00	0.00
			29	0.28	1.17	0.00	0.00	0.00	0.00	0.00	0.00	0.00	0.00	0.00	0.00	0.00	0.00	0.00
			36	0.08	0.82	0.00	0.00	0.00	0.00	0.00	0.00	0.00	0.00	0.00	0.00	0.00	0.00	0.00
43	0.01		0.11	0.00	0.00	0.00	0.00	0.00	0.00	0.00	0.00	0.00	0.00	0.00	0.00	0.00
50	0.00		0.00	0.00	0.00	0.00	0.00	0.00	0.00	0.00	0.00	0.00	0.00	0.00	0.00	0.00

**Table 29:** Composition of flue gases during PE combustion in a fluidised bed of Fe_2_O_3_-cenospheres at 900 °C.

Temp.	Polymer	Mass	Time	CO2	CO	CH4	C2H6	C3H8	C2H4	C4+	HCHO	CH3CHO	Acrolein	C2H2	C6H6	CH3OH	CH3COOH	CH3COCH3
°C	-	mg	s	%_vol._	ppm													
900	PE	200	0	0.00	0.00	0.00	0.00	0.00	0.00	0.00	0.00	0.00	0.00	0.00	0.00	0.00	0.00	0.00
			7	1.26	2.66	0.00	0.00	0.00	0.00	0.00	0.00	0.00	0.00	0.00	0.00	0.00	0.00	0.00
			14	3.28	20.83	0.00	0.00	0.00	0.00	0.00	0.00	0.00	0.00	0.00	0.00	0.00	0.00	0.00
			21	1.07	8.11	0.00	0.00	0.00	0.00	0.00	0.00	0.00	0.00	0.00	0.00	0.00	0.00	0.00
			28	0.33	1.48	0.00	0.00	0.00	0.00	0.00	0.00	0.00	0.00	0.00	0.00	0.00	0.00	0.00
			35	0.10	0.00	0.00	0.00	0.00	0.00	0.00	0.00	0.00	0.00	0.00	0.00	0.00	0.00	0.00
			42	0.00	0.00	0.00	0.00	0.00	0.00	0.00	0.00	0.00	0.00	0.00	0.00	0.00	0.00	0.00

		202	0	0.00	0.00	0.00	0.00	0.00	0.00	0.00	0.00	0.00	0.00	0.00	0.00	0.00	0.00	0.00
			7	1.16	10.56	2.11	0.00	0.00	0.00	0.00	0.00	0.00	0.00	0.00	0.00	0.00	0.00	0.00
			15	2.72	212.44	32.34	0.00	0.00	0.00	0.00	0.00	0.00	0.00	0.00	0.00	0.00	0.00	0.00
			22	0.96	52.45	9.98	0.00	0.00	0.00	0.00	0.00	0.00	0.00	0.00	0.00	0.00	0.00	0.00
			29	0.30	16.35	2.65	0.00	0.00	0.00	0.00	0.00	0.00	0.00	0.00	0.00	0.00	0.00	0.00
			36	0.09	4.18	1.77	0.00	0.00	0.00	0.00	0.00	0.00	0.00	0.00	0.00	0.00	0.00	0.00
			43	0.02	1.74	0.23	0.00	0.00	0.00	0.00	0.00	0.00	0.00	0.00	0.00	0.00	0.00	0.00

		100	0	0.00	0.00	0.00	0.00	0.00	0.00	0.00	0.00	0.00	0.00	0.00	0.00	0.00	0.00	0.00
			7	0.18	1.45	0.00	0.00	0.00	0.00	0.00	0.00	0.00	0.00	0.00	0.00	0.00	0.00	0.00
			15	2.16	1.75	0.00	0.00	0.00	0.00	0.00	0.00	0.00	0.00	0.00	0.00	0.00	0.00	0.00
			22	0.91	0.52	0.00	0.00	0.00	0.00	0.00	0.00	0.00	0.00	0.00	0.00	0.00	0.00	0.00
			29	0.29	0.00	0.00	0.00	0.00	0.00	0.00	0.00	0.00	0.00	0.00	0.00	0.00	0.00	0.00
			36	0.09	0.00	0.00	0.00	0.00	0.00	0.00	0.00	0.00	0.00	0.00	0.00	0.00	0.00	0.00
43	0.02		0.00	0.00	0.00	0.00	0.00	0.00	0.00	0.00	0.00	0.00	0.00	0.00	0.00	0.00
50	0.00		0.00	0.00	0.00	0.00	0.00	0.00	0.00	0.00	0.00	0.00	0.00	0.00	0.00	0.00

**Table 30:** Composition of flue gases during LLDPE combustion in a fluidised bed of Fe_2_O_3_-cenospheres at 900 °C.

Temp.	Polymer	Mass	Time	CO2	CO	CH4	C2H6	C3H8	C2H4	C4+	HCHO	CH3CHO	Acrolein	C2H2	C6H6	CH3OH	CH3COOH	CH3COCH3
°C	-	mg	s	%_vol._	ppm													
900	LLDPE	200	0	0.00	0.00	0.00	0.00	0.00	0.00	0.00	0.00	0.00	0.00	0.00	0.00	0.00	0.00	0.00
			7	1.93	4.92	0.00	0.00	0.00	0.00	0.00	0.00	0.00	0.00	0.00	0.00	0.00	0.00	0.00
			14	3.21	6.42	0.00	0.00	0.00	0.00	0.00	0.00	0.00	0.00	0.00	0.00	0.00	0.00	0.00
			21	1.02	3.28	0.00	0.00	0.00	0.00	0.00	0.00	0.00	0.00	0.00	0.00	0.00	0.00	0.00
			28	0.33	1.19	0.00	0.00	0.00	0.00	0.00	0.00	0.00	0.00	0.00	0.00	0.00	0.00	0.00
			35	0.10	1.01	0.00	0.00	0.00	0.00	0.00	0.00	0.00	0.00	0.00	0.00	0.00	0.00	0.00
			42	0.04	1.30	0.00	0.00	0.00	0.00	0.00	0.00	0.00	0.00	0.00	0.00	0.00	0.00	0.00
			50	0.00	0.00	0.00	0.00	0.00	0.00	0.00	0.00	0.00	0.00	0.00	0.00	0.00	0.00	0.00

		205	0	0.00	0.00	0.00	0.00	0.00	0.00	0.00	0.00	0.00	0.00	0.00	0.00	0.00	0.00	0.00
			7	0.31	0.00	0.00	0.00	0.00	0.00	0.00	0.00	0.00	0.00	0.00	0.00	0.00	0.00	0.00
			14	4.08	0.00	0.00	0.00	0.00	0.00	0.00	0.00	0.00	0.00	0.00	0.00	0.00	0.00	0.00
			21	1.99	0.00	0.00	0.00	0.00	0.00	0.00	0.00	0.00	0.00	0.00	0.00	0.00	0.00	0.00
			28	0.63	0.00	0.00	0.00	0.00	0.00	0.00	0.00	0.00	0.00	0.00	0.00	0.00	0.00	0.00
			36	0.20	0.00	0.00	0.00	0.00	0.00	0.00	0.00	0.00	0.00	0.00	0.00	0.00	0.00	0.00
			43	0.05	0.00	0.00	0.00	0.00	0.00	0.00	0.00	0.00	0.00	0.00	0.00	0.00	0.00	0.00
			50	0.01	0.00	0.00	0.00	0.00	0.00	0.00	0.00	0.00	0.00	0.00	0.00	0.00	0.00	0.00
			57	0.00	0.00	0.00	0.00	0.00	0.00	0.00	0.00	0.00	0.00	0.00	0.00	0.00	0.00	0.00

		98	0	0.00	0.00	0.00	0.00	0.00	0.00	0.00	0.00	0.00	0.00	0.00	0.00	0.00	0.00	0.00
			7	0.59	0.00	0.00	0.00	0.00	0.00	0.00	0.00	0.00	0.00	0.00	0.00	0.00	0.00	0.00
			14	2.19	0.00	0.00	0.00	0.00	0.00	0.00	0.00	0.00	0.00	0.00	0.00	0.00	0.00	0.00
			21	0.80	0.00	0.00	0.00	0.00	0.00	0.00	0.00	0.00	0.00	0.00	0.00	0.00	0.00	0.00
			29	0.25	0.00	0.00	0.00	0.00	0.00	0.00	0.00	0.00	0.00	0.00	0.00	0.00	0.00	0.00
			36	0.07	0.00	0.00	0.00	0.00	0.00	0.00	0.00	0.00	0.00	0.00	0.00	0.00	0.00	0.00
43	0.01		0.00	0.00	0.00	0.00	0.00	0.00	0.00	0.00	0.00	0.00	0.00	0.00	0.00	0.00
50	0.00		0.00	0.00	0.00	0.00	0.00	0.00	0.00	0.00	0.00	0.00	0.00	0.00	0.00	0.00

**Table 31:** Composition of flue gases during HDPE combustion in a fluidised bed of Fe_2_O_3_-cenospheres at 900 °C.

Temp.	Polymer	Mass	Time	CO2	CO	CH4	C2H6	C3H8	C2H4	C4+	HCHO	CH3CHO	Acrolein	C2H2	C6H6	CH3OH	CH3COOH	CH3COCH3
°C	-	mg	s	%_vol._	ppm													
900	HDPE	205	0	0.00	0.00	0.00	0.00	0.00	0.00	0.00	0.00	0.00	0.00	0.00	0.00	0.00	0.00	0.00
			7	0.62	0.46	0.00	0.00	0.00	0.00	0.00	0.00	0.00	0.00	0.00	0.00	0.00	0.00	0.00
			14	3.12	1.77	0.00	0.00	0.00	0.00	0.00	0.00	0.00	0.00	0.00	0.00	0.00	0.00	0.00
			21	2.06	0.82	0.00	0.00	0.00	0.00	0.00	0.00	0.00	0.00	0.00	0.00	0.00	0.00	0.00
			28	0.68	0.50	0.00	0.00	0.00	0.00	0.00	0.00	0.00	0.00	0.00	0.00	0.00	0.00	0.00
			35	0.22	0.16	0.00	0.00	0.00	0.00	0.00	0.00	0.00	0.00	0.00	0.00	0.00	0.00	0.00
			42	0.07	0.00	0.00	0.00	0.00	0.00	0.00	0.00	0.00	0.00	0.00	0.00	0.00	0.00	0.00
			50	0.02	0.00	0.00	0.00	0.00	0.00	0.00	0.00	0.00	0.00	0.00	0.00	0.00	0.00	0.00

		301	0	0.00	0.00	0.00	0.00	0.00	0.00	0.00	0.00	0.00	0.00	0.00	0.00	0.00	0.00	0.00
			7	0.10	0.00	0.00	0.00	0.00	0.00	0.00	0.00	0.00	0.00	0.00	0.00	0.00	0.00	0.00
			14	3.81	6.81	0.00	0.00	0.00	0.00	0.00	0.00	0.00	0.00	0.00	0.00	0.00	0.00	0.00
			21	4.18	13.43	0.00	0.00	0.00	0.00	0.00	0.00	0.00	0.00	0.00	0.00	0.00	0.00	0.00
			28	1.62	3.35	0.00	0.00	0.00	0.00	0.00	0.00	0.00	0.00	0.00	0.00	0.00	0.00	0.00
			36	0.52	0.00	0.00	0.00	0.00	0.00	0.00	0.00	0.00	0.00	0.00	0.00	0.00	0.00	0.00
			43	0.16	0.00	0.00	0.00	0.00	0.00	0.00	0.00	0.00	0.00	0.00	0.00	0.00	0.00	0.00
			50	0.06	0.00	0.00	0.00	0.00	0.00	0.00	0.00	0.00	0.00	0.00	0.00	0.00	0.00	0.00

		100	0	0.00	0.00	0.00	0.00	0.00	0.00	0.00	0.00	0.00	0.00	0.00	0.00	0.00	0.00	0.00
			7	0.33	2.13	0.00	0.00	0.00	0.00	0.00	0.00	0.00	0.00	0.00	0.00	0.00	0.00	0.00
			14	2.05	1.33	0.00	0.00	0.00	0.00	0.00	0.00	0.00	0.00	0.00	0.00	0.00	0.00	0.00
			21	1.05	0.27	0.00	0.00	0.00	0.00	0.00	0.00	0.00	0.00	0.00	0.00	0.00	0.00	0.00
			28	0.34	0.00	0.00	0.00	0.00	0.00	0.00	0.00	0.00	0.00	0.00	0.00	0.00	0.00	0.00
			36	0.10	0.00	0.00	0.00	0.00	0.00	0.00	0.00	0.00	0.00	0.00	0.00	0.00	0.00	0.00
43	0.03		0.00	0.00	0.00	0.00	0.00	0.00	0.00	0.00	0.00	0.00	0.00	0.00	0.00	0.00
50	0.00		0.00	0.00	0.00	0.00	0.00	0.00	0.00	0.00	0.00	0.00	0.00	0.00	0.00	0.00

## 3 Experimental design, materials, and methods

Fluidised bed reactor with an inner diameter of 96 mm was filled by 300 g cenospheres. Two kinds of cenospheric beds were tested. Inert cenospheres - a fraction of raw cenospheres but selected so that the shells of the spheres have not broken and perforated surfaces. Catalytic cenospheres were covered by Fe_2_O_3_ (12,5%_mass_). Inert and catalytic cenospheres have diam. 0.25-0.3 mm. Bed materials were introduced into fluidised bed by air. Thermal degradation of polyethylene samples was carried out at five bed temperatures: from 500 to 900°C, at 100°C intervals. Three types of polyethylene samples were tested: PE, HDPE, LLDPE. Samples (≈ 200 mg) were thrown from the top of the reactor. Video recording of the bed surface and audio analysis have proven polymer sinking and degradation inside the bed. Released gases were sampled by a probe form freeboard zone. The gas path was heated to 180°C. Infrared analyses were performed on-line during fluidised bed combustion. The recording of the spectrum and its deconvoluting took 7-8 seconds. Calcmet software (Gasmet Technologies Oy) uses a modified least-squares method to determine the appropriate concentrations of compounds in the sample based on a set of reference spectra. This method is modified by additional masking of certain ranges of reference spectra. The analyzes were performed based on the following reference compound spectra (with detailed analysis ranges of wavenumber): H_2_O (3400 - 3200 cm^−1^); CO_2_ 3257-3723 cm^−1^); CO (2000-2200 cm^−1^ and 2540-2590 cm^−1^); methane (2600-3200 cm^−1^); ethane (2600-3200 cm^−1^); propane (2600-3200 cm^−1^); ethylene (910-1150 cm^−1^); hexane (2600-3200 cm^−1^); formaldehyde (2550-2850 cm^−1^); acetaldehyde (2550-2950 cm^−1^); acrolein (2550-2900 cm^−1^); acetylene 2995-3412 cm^−1^); benzene (2856-3226 cm^−1^); methanol 910-1150 cm^−1^ and 2550-2920 cm^−1^); formic acid (895-1192 cm^−1^); acetic acid (895-1335 cm^−1^); acetone (895-1380 cm^−1^). Selected wavenumbers indicated characteristic absorbance bands of given compounds resulting from its specific structure. The deconvolution method for the quantification of the concentration of compounds in the flue gases during the FTIR analysis was explained in the supplementary material for the article [10]. The Calcmet software collects, stores spectra of the sample gas, and analyzes the concentrations of the flue gas components. The apparatus performed 50 scans per second in the range of wavenumber 900–4200 cm^−1^. The results of the analysis were copied from the Calcmet text file and were pasted into the manuscript as tables. Columns referring to undetected compounds were removed. Concentration changes over time are summarized in Table 2, Table 3, Table 4, Table 5, Table 6, Table 7, Table 8, Table 9, Table 10, Table 11, Table 12, Table 13, Table 14, Table 15, Table 16 and Table 17, Table 18, Table 19, Table 20, Table 21, Table 22, Table 23, Table 24, Table 25, Table 26, Table 27, Table 28, Table 29, Table 30, Table 31, respectively, for inert and catalytic bed.

## Declaration of Competing Interest

The authors declare that they have no known competing financial interests or personal relationships which have, or could be perceived to have, influenced the work reported in this article.

## Appendix Supplementary materials

Image, application 1

Image, application 2
